# Glioblastoma Cell Migration, Invasion and Vasculogenic Mimicry Downmodulated by Novel uPAcyclin Derivatives

**DOI:** 10.3390/cells14040259

**Published:** 2025-02-12

**Authors:** Federica Santoro, Francesco Merlino, Diego Brancaccio, Iolanda Camerino, Stefania Belli, Amelia Cimmino, Paolo Grieco, Luca Colucci-D’Amato, Maria Patrizia Stoppelli, Paola Franco, Alfonso Carotenuto

**Affiliations:** 1 Department of Pharmacy, University of Naples Federico II, 80131 Naples, Italy; federica.santoro@unina.it (F.S.); francesco.merlino@unina.it (F.M.); diego.brancaccio@unina.it (D.B.); paolo.grieco@unina.it (P.G.); 2 Centro Interuniversitario di Ricerca sui Peptidi Bioattivi “Carlo Pedone” (CIRPeB), University of Naples Federico II, 80134 Naples, Italy; 3 Department of Environmental, Biological and Pharmaceutical Sciences and Technologies, University of Campania “Luigi Vanvitelli”, 81100 Caserta, Italy; iolanda.camerino@unicampania.it (I.C.); luca.colucci@unicampania.it (L.C.-D.); 4 Institute of Genetics and Biophysics “A. Buzzati Traverso” (IGB-ABT), National Research Council, 80131 Naples, Italy; stefania.bell21@gmail.com (S.B.); amelia.cimmino@igb.cnr.it (A.C.); mariapatrizia.stoppelli@unicamillus.org (M.P.S.); paola.franco@igb.cnr.it (P.F.); 5 InterUniversity Center for Research in Neurosciences (CIRN), 80131 Naples, Italy; 6 Departmental Faculty of Medicine and Surgery, UniCamillus-Saint Camillus International University of Health Sciences, 00131 Rome, Italy

**Keywords:** glioblastoma, cyclic peptides, vasculogenic mimicry, tumor migration, tumor invasion

## Abstract

Despite extensive efforts to develop new treatments, the prognosis for glioblastoma multiforme (GBM) is extremely unfavorable, urging the identification of new chemotherapeutics. A previous work identified the cyclic decapeptide uPAcyclin as a potent inhibitor of GBM cell migration, matrix invasion and vascular-like structures’ formation, acting through binding to αV integrins and not interfering with cell proliferation or survival. These clearcut activities prompted us to design and test novel derivatives on cultured U87-MG and U251 GBM-MG human cells. With the exception of the residues involved in peptide cyclization, residues were Ala-substituted one by one and the single peptides tested for binding affinity for the αV target integrin, the inhibition of migration, invasion and vasculogenic mimicry. The first screening highlighted peptides with a low binding affinity and low inhibitory ability (Ala4,7,9 derivatives) and peptides with affinity and inhibitory capacity higher than uPAcyclin (Ala2,5,6,8 derivatives). The integration of these results with conformational studies led to the design of the di-substituted variant uPAcyclin. Intriguingly, at least ten-fold greater anti-migratory and anti-invasive effects of the [Ala^2^,Ala^5^]uPAcyclin variant compared to uPAcyclin were found. The latter variant also exhibited a greater inhibitory potential for vascular-like structures’ formation by matrix-seeded GBM cells. These studies shed light on the functional relevance of single amino acid residues in uPAcyclin and lead to the identification of therapeutically interesting new variants as promising candidates for anti-GBM therapies.

## 1. Introduction

Glioblastoma (GBM) is the most common malignant primary brain tumor, characterized by micro-vascular proliferation, cellular necrosis and the pronounced invasiveness of surrounding parenchima with high recurrency and a high mortality rate [1,2]. GBM disease shows a rapid expansion with the disruption of brain structures, neurological symptoms and signs of intracranial pressure. Despite extended surgical resection, as well as radio- and chemotherapy, overall survival has not significantly improved in the last decade [3]. In particular, surgery still represents an important pillar of treatment since the extent of resection significantly improves survival rates [4]. Nevertheless, the infiltrative behavior of GBM poses considerable challenges, with tumor cells spreading into healthy brain tissue. Therefore, a total resection, without damaging essential brain areas, potentially resulting in postoperative neurological impairment is difficult to achieve [5]. Moreover, GBM stem cells, promoting radioresistance, strongly limit the effects of radiotherapy [6]. However, a multimodal approach is currently the first-line, standard therapy based on the crucial phase 3 clinical trial published by Stupp and colleagues [7]. In this study, surgical resection is followed by radiotherapy and concomitant chemotherapy with Temozolomide, an alkylating drug that extends the median overall survival from 12.1 months to 14.6 months. The use of antiangiogenic therapy with Bevacizumab, a humanized monoclonal antibody targeting VEGF, extended progression-free survival (PFS), although it was linked to greater toxicity and showed no benefit in overall survival [8]. Moreover, vasculogenic mimicry (VM) has emerged as a new mechanism accounting for the aggressiveness and resistance to therapy of different types of cancers, including GBM. Although its cellular and molecular bases are still not entirely understood, VM basically involves the formation of vessel-like structures from cancer cells [9]. In particular, cancer stem cells are believed to contribute to VM formation [10]. Thus, therapeutic options are limited and inefficacious, and they produce harmful side effects, urging the need for new pharmacological approaches.

Among recognized target molecules overexpressed in GBM, there are members of the integrin family, known to be crucial to GBM local invasion. Because their impairment affects tumor proliferation and invasion, integrins are regarded as targets for innovative therapies [11]. The upregulation of integrins occurs in tumor and stromal cells, and among them, the expression of αVβ3 integrin, also known as the vitronectin receptor, correlates with high-grade GBM. Therefore, antagonists of αVβ3 are attractive therapeutic candidates to counteract GBM tumors [12]. GBM invasion is supported by an interplay of factors contributing to malignancy, including mitochondria dysfunction, which may contribute to tumorigenesis through the humanin-dependent α*V* (ITGAV)-TGFβ signaling axis [13]. Recent evidence shows that GBM cell migration through brain tissues requires CD44, myosin and integrins, playing a crucial role in the motor-clutch adhesive mechanism [14]. Integrins are a family of extracellular matrix (ECM) receptors, each formed by one α and one β subunit, with fundamental functions in all higher organisms. They represent a connection between ECM molecules and the cell cytoskeleton, activating transmembrane signaling pathways that govern cell shape, proliferation, migration and adhesion. The most known cell recognition minimal motif common to cell adhesive ECM proteins is the Arg-Gly-Asp (RGD) sequence, which occurs in most integrin ligands. A large body of information, together with the increasingly recognized role of integrins in GBM and other malignancies, led to the design of multiple peptidic RGD-based ligands, which were widely employed to target integrin-overexpressing tumors, mostly as anti-angiogenetic inhibitors [15]. A known integrin antagonist is the cyclic RGD pentapeptide named Cilengitide, the first anti-angiogenic small molecule targeting the integrins αVβ3, αVβ5, and α5β1. This drug was administered to patients with newly diagnosed glioblastoma and a methylated MGMT promoter, subjected to radiotherapy and concomitant Temozolomide chemotherapy, but it yielded disappointing results [16]. In general, the clinical development of anti-integrin inhibitors, especially those related to the RGD-binding subfamily of αV integrins, has been attempted for osteoporosis, as well as ophthalmic and fibrotic diseases, resulting in fewer than a dozen approved integrin-targeting drugs, whereas others are in trial for glioma, multiple myeloma, and non-tumoral pathologies like ulcerative colitis, liver fibrosis and age-related macular degeneration [17]. Although integrins are still regarded as important targets in a variety of diseases, the design of anti-integrin drugs should explore new modalities and learn from previous experience to progress toward novel and efficacious therapeutic strategies.

Previous work from this group reported an RGD-independent novel inhibitor of αV integrins’ activity, named uPAcyclin, a cyclic decapeptide, active to prevent lung metastases in a mouse model of tumor dissemination [18]. This peptide is derived from a non-catalytic region of urokinase (uPA), a serine protease cleaving plasminogen, and thus generates the broad-spectrum plasmin serine protease, mostly active on ECM components. The uPA has catalytically independent motogen activity that derives from its amino-terminal growth factor domain (GFD, residues 1–49), binding to the urokinase receptor or uPAR, and its connecting peptide region (CP, residues 132–158) [19]. The CP region binds to αV integrins, bridging uPAR and the αVβ5 and eliciting migratory signaling [20].

The CP region exerts a dual activity on cell migration that is retained by the derived peptides: the amino-terminal end performs a clearcut inhibitory function, whereas the carboxy-terminal end is endowed with a stimulatory ability [21]. Our previous work highlighted a phosphorylation site on Ser138/139, conferring remarkable anti-migratory properties to the full uPA, which are retained by the S138E/S303E mutations [22]. Within the N-terminal CP region, the Å6 octapeptide, corresponding to uPA residues 136–143 (Ac-K^136^PSSPPEE^143^-NH_2_), is endowed with clearcut antiangiogenetic and anti-metastatic activities in mice [23]. The Å6 was tested in gynecologic malignancies, and phase I trials indicated that it was well tolerated. Further phase II studies indicated a delayed disease progression in treated individuals [24,25].

These findings, together with structural analyses, suggested the design of new peptides, uPAcyclin and its linear analog (Table 1), corresponding to the *N*-terminal region of CP, incorporating the S138E substitution and stabilizing the putative bioactive conformation [18]. The two decapeptides bind with high affinity to the αV-integrins and markedly inhibit the migration and invasion of HT1080 fibrosarcoma and MDA-MB-231 breast carcinoma cells. Also, they induced a partial reversion of the cancer-associated fibroblasts (CAFs) phenotype and markedly reduced the pro-invasive ability of peritumoral CAFs from breast cancer patients. Noticeably, the daily intravenous administration of uPAcyclin strongly reduces lung dissemination in nude mice injected with fibrosarcoma cells.

More recent work established that nanomolar concentrations of uPAcyclin counteract the migration, invasion and vascular structure formation of GBM cells, raising the possibility of a therapeutic use in this deadly tumor [26]. The relevance of invasiveness and neo-angiogenesis in the pathophysiology and aggressiveness of GBM prompted us to further study the uPAcyclin, investigating the role of individual residues in the overall inhibitory activity; thus, a thorough study on the structure and functional properties of uPAcyclin via NMR analyses and functional assays is reported. These results not only increase our knowledge of the three-dimensional uPAcyclin structure but also show that the binding ability and biological activity of this cyclic peptide can be modulated independently. Finally, these data uncovered Ala analogs of uPAcyclin endowed with a 100-fold increased affinity and biological activities, with measurable activity in the low picomolar range.

## 2. Materials and Methods

### 2.1. Cell Lines and Culture Conditions

The U87-MG and U251-MG human glioblastoma cell lines were cultured as reported in [26].

### 2.2. Peptide Synthesis

Peptide sequences were synthesized using the Fmoc-based ultrasonication-assisted solid-phase peptide synthesis (US-SPPS) method [27]. The synthesis began with the Fmoc Rink amide resin (0.1 mmol; 0.72 mmol/g as loading, 100–200 mesh as particle size), which was swollen in DMF for 20 min. To remove the Fmoc protecting group, a 20% piperidine solution in DMF was added, and the mixture was subjected to ultrasonic irradiation (0.5 + 1 min) in a bath, ensuring that the water level covered the reaction mixture. After each step, the resin was filtered and washed (3 × 2 mL of DMF). For coupling reactions, a solution containing the Fmoc-amino acid (2 equiv), COMU (2 equiv), Oxyma (2 equiv), and DIEA (4 equiv) in DMF was prepared. The resin was exposed to ultrasonic irradiation for 5 min to promote coupling. The *N*-terminal ends of all uPAcyclins were acetylated using a solution of Ac_2_O (2 equiv) and DIPEA (4 equiv) in DMF, with shaking on an automated shaker for 30 min at rt. For FITC-uPAcyclin the *N*-terminal was labeled with fluorescein isothiocyanate (FITC) prior to introducing a spacer. In order to monitor Fmoc deprotection and coupling, colorimetric Kaiser or Chloranil tests were employed to detect solid-phase bound primary and secondary amines, respectively. Once peptide elongation was complete, allyl-based protecting groups on Glu and Lys residues were selectively removed, as described previously [28,29]. In brief, the resin was washed with DCM (3 × 2 mL) and treated with a solution of Pd(PPh_3_)_4_ (0.15 equiv) and NDMBA (3 equiv) in a DCM/DMF mixture (3:2, *v/v*) under gentle shaking for 1 h under Ar. The resin was then filtered, washed (3 × 2 mL DMF, 3 × 2 mL DCM), and dried. This procedure was repeated once more. Subsequently, the resin was washed with a 0.5% solution of sodium *N,N*-diethyldithiocarbamate in DMF (30 min × 2), and the complete removal of the allyl groups was confirmed via LC-MS analysis. For cyclization, the resulting amine and carboxylic acid groups were coupled using PyAOP (2 equiv), HOAt (2 equiv), and DIEA (4 equiv) in a DMF/DCM mixture (1:1, *v/v*). The reaction was allowed to proceed for 16 h at rt, and conversion to the cyclic product was monitored via LC-MS analysis. The peptidyl resin was then dried under a vacuum, and the peptides were cleaved using a 95% TFA solution. The cleaved peptides were precipitated with Et_2_O and centrifuged (6000 rpm × 15 min), and the supernatants were discarded. The resulting amorphous solids were dried and dissolved in water/acetonitrile (9:1) mixture for reverse-phase HPLC analysis.

The purification of peptides was conducted using RP-HPLC (Preparative Liquid Chromatograph LC-8A from Shimadzu, Kyoto, Japan) equipped with a preparative column (Phenomenex Kinetex C18 column, 5 μm, 100 Å, 150 × 21.2 mm). Linear gradients of MeCN (0.1% TFA) in water (0.1% TFA) were applied (10–90% over 30 min) with a flow rate of 10 mL/min and UV detection at 220 nm. The purified peptides were obtained by lyophilizing appropriate fractions after MeCN removal through rotary evaporation. Analytical UHPLC (Liquid Chromatograph LC-30AD Nexera from Shimadzu, Kyoto, Japan) was used to assess peptide purity, employing a Phenomenex Kinetex reversed-phase column (C18, 5 μm, 100 Å, 150 × 4.6 mm). A gradient of MeCN (0.1% TFA) in water (0.1% TFA) was used (10–90% over 15 min) at a flow rate of 1 mL/min with UV detection at 220 nm. Peptides intended for biological assays were purified to >95% purity, and their molecular ions were confirmed via mass spectrometry prior to use.

### 2.3. NMR Conformational Analysis

NMR samples were prepared by dissolving selected peptides in 0.54 mL of H_2_O and 0.06 mL of ^2^H_2_O (pH 5.5) to obtain a 1 mM final concentration. NMR spectra were recorded on a Bruker Avance NEO 600 MHz spectrometer equipped with a z-gradient 5 mm triple-resonance probe head at 25 °C. One-dimensional (1D) NMR spectra were recorded in the Fourier mode with quadrature detection. 2D DQF-COSY [30,31], TOCSY [32] and NOESY [33] spectra (Supporting Information, Figures S1–S3) were recorded in the phase-sensitive mode using the method from States [34]. Further acquisition details are reported in [18]. The qualitative and quantitative analyses of 2D NMR spectra were obtained using the program XEASY [35]. Complete ^1^H NMR chemical shift assignments were achieved for [Ala^5^]uPAcyclin, [Ala^2^,Ala^5^]uPAcyclin and for the main conformer of [Ala^1^]uPAcyclin according to the Wüthrich procedure [36] with the support of the CARA software package, version 1.5.5 (Supporting Information, Tables S1–S4). The NOE-based distance restraints were obtained from NOESY spectra (Supporting Information, Tables S5 and S6) and used as constraints in the simulated annealing calculations using the program CYANA, version 3.98 [37]. Only NOE-derived constraints were considered in the annealing procedures (Supporting Information, Tables S5 and S6). The best 10 structures from the annealing procedure were optimized using the Discover algorithm (Accelrys, San Diego, CA, USA) and the consistent valence force field [38]. The minimization lowered the total energy of the structures; no residue was found in the disallowed region of the Ramachandran plot. Molecular graphics images were realized using the UCSF Chimera package, version 1.18 [39].

### 2.4. Binding Assay

The U87-MG and U251-MG cell lines were harvested and acid-treated, as described [40]. Briefly, 2 × 10^6^ cells/sample were pre-incubated for 30 min at 4 °C with uPAcyclin and Ala peptides and subsequently exposed to 50 nM FITC-uPAcyclin for 2 h at 4 °C, as described [26]. The experiments were performed three times, with duplicate samples, and analyzed as reported in the statistical analysis.

### 2.5. Migration and Invasion Assays

The migration and invasion assays were performed with Boyden chambers, according to De Vincenzo et al. [41], using 11 × 10^4^ U87-MG and U251-MG glioblastoma cells/sample pre-treated for 40 min at 37 °C with the indicated effectors and then allowed to migrate for 3 h or to invade for 5 h at 37 °C toward 5% FBS in DMEM/0.1% BSA. The migrated or invaded cells were reported as a percentage of basal random migration or invasion and analyzed as reported in the Statistical Analyses section. The migration and invasion experiments were conducted in triplicate.

### 2.6. Analysis of Cytoskeleton

The U87-MG cells were analyzed for their cytoskeletal organization upon exposure to the indicated peptides. Briefly, 2 × 10^5^ cells/sample were harvested via mild trypsinization, pre-treated for 40 min at 37 °C with 100 nM of selected peptides, and incubated with 5% FBS for 3 h at 37 °C. Samples diluted in a serum-free medium were included as negative controls. Then cells were fixed, permeabilized, and stained with 0.1 μg/mL rhodamine–phalloidin (Sigma-Merck, St. Louis, MO, USA) for 40 min at rt. To stain nuclei, the slides were incubated with 20 ng/mL DAPI (4′,6-Diamidino-2- phenylindole, Sigma-Merck, St. Louis, MO, USA) for 10 min at rt, mounted with Mowiol Mounting Medium (Sigma-Merck, St. Louis, MO, USA), and examined with the DMI6000 Leica Inverted Fluorescence Microscope (Zeiss, Milan, Italy) at 40× magnification. At least 4 fields/samples were acquired, and the total fluorescence was measured with ImageJ.

### 2.7. Vasculogenic Mimicry Assay

The tube formation assay was performed as previously described by Franco et al. [26], using 3 × 10^4^ U87-MG and 1.5 × 10^4^ U251 cells/sample in the absence or presence of specific effectors included at time 0. The evaluation of vascular-like structures, as the branching points number [42], was performed using the Inverted Microscope DMI Leica 6000 (Zeiss, Milan, Italy) and assessed in 4/5 random fields using the ImageJ 1.52a Software.

### 2.8. Cell Viability Assay

Cell viability was tested in DMEM/10% FBS in the presence of 100 nM uPAcyclin or Ala peptides via a Trypan blue exclusion assay, as previously reported [26], using 4 × 10^4^ U87-MG and U251-MG glioblastoma cells/sample followed for 24, 48 and 72 h.

### 2.9. Statistical Analyses

Unless otherwise specified, experiments were performed, at least, three times in triplicate and expressed as the means ± standard deviations, indicated by error bars. Differences between data sets were determined via Student’s *t* test. Differences described as significant are indicated in the figures with *p*-values ≤ 0.05 (*), ≤ 0.005 (**) or ≤ 0.001 (***).

## 3. Results

The interesting activities of uPAcyclin prompted us to develop some analogs by changing the residues one by one with alanine (Ala-scan, Table 1).

### 3.1. Specific Binding and Biological Properties of All Ala Peptides

**Figure 1:**
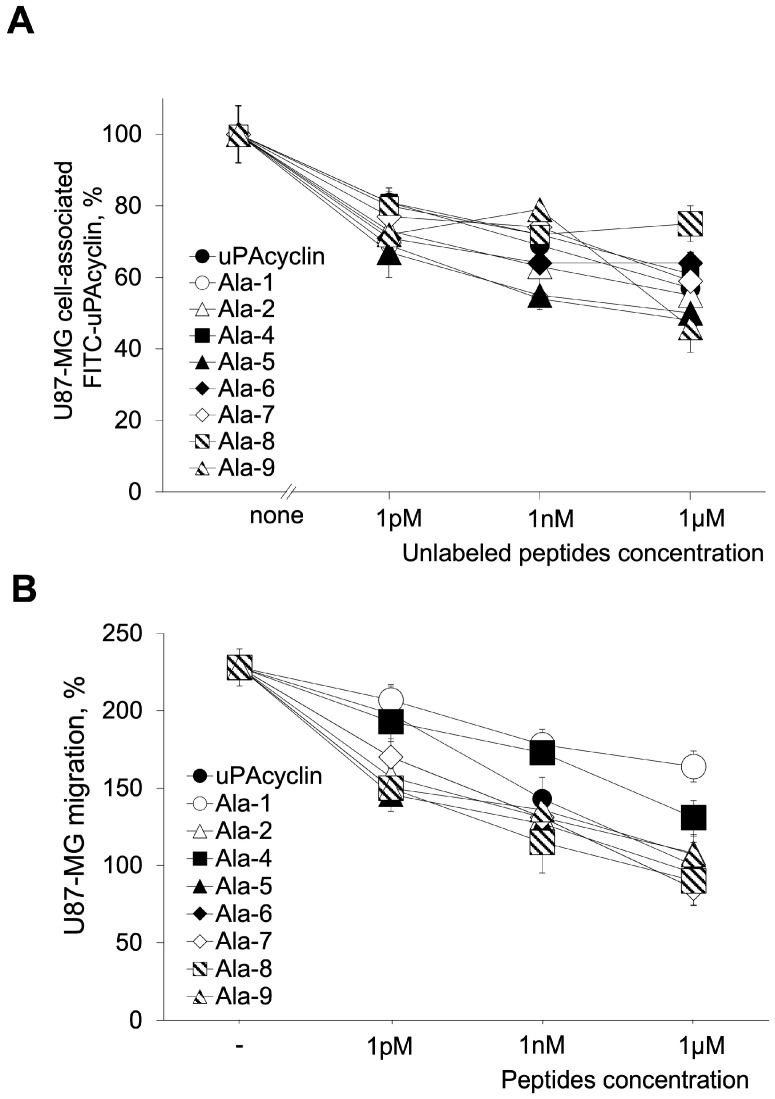
Binding of Ala peptides to U87-MG cells and effect of uPAcyclin and Ala peptides on U87-MG cellular migration. (**A**) A total of 2 × 10^6^ U87-MG cells were pre-incubated for 30 min at 4 °C with increasing concentrations of unlabeled uPAcyclin and Ala peptides and then exposed to 50 nM of FITC-uPAcyclin for 2 h at 4 °C. Cell surface-associated fluorescence, as percentage of the sample with no FITC-uPAcyclin, is shown. Experiments were carried out in duplicate, and the data shown here are representative of three independent experiments. (**B**) 11 × 10^4^ U87-MG cells/sample were pre-treated for 40 min at 37 °C with increasing concentrations of uPAcyclin or the indicated Ala peptides and then assayed in Boyden chambers for 5% FBS-dependent migration for 3 h at 37 °C. At the end of incubation, the filters were processed as described in the Methods section. For clarity here and in the following Figures, Ala-x indicates the peptide with substituted x-position. For both assays, the *p*-values are in the range of 0.005 ≤ *p* ≤ 0.001, apart from [Ala1]uPAcyclin in the migration assay with *p* ≤ 0.05.

Previous work showed the specific interaction of FITC-conjugated uPAcyclin to both U87-MG and U251-MG cell lines using embryonic kidney cells over-expressing αV-integrin subunit (HEK-293/αV) and parental HEK-293 as controls [26]. This assay confirmed that FITC-uPAcyclin binds specifically to both GBM cell lines and to the HEK-293/αV, whereas little interaction is detected with HEK-293 cells (Supporting Information, Table S7). All GBM cells express a comparable level of αV, and the inhibitory effect of uPAcyclin is αV-dependent [26]. To investigate the ability of novel Ala-scan peptides to compete with uPAcyclin for binding to integrin αV receptors on the GBM cell surface, the specific interaction of FITC-conjugated uPAcyclin with U87-MG living cells was tested via a receptor binding assay. U87-MG GBM cells were pre-incubated with increasing concentrations of unlabeled Ala peptides and then exposed to FITC-uPAcyclin (Figure 1A). As expected, uPAcyclin shows a dose-dependent ability to abolish the binding of FITC-uPAcyclin, with a Kd_app_ of around 100 pM. When considering the relative Kd_app_ of uPAcyclin variants, it can be inferred that the replacement either of each of the three prolines (Pro^2^, Pro^5^, Pro^6^) or Lys^1^ increases the binding affinity of the resulting peptide compared to uPAcyclin. In contrast, the Ala mutation of other residues is detrimental to the peptide binding affinity. In the displacement assay, a comparison of the relative Kd_app_ exhibited via the tested peptides yielded the following scale, averaged across different peptide concentrations: [Ala^4^] ≈ [Ala^8^] ≥ [Ala^7^] ≥ uPAcyclin ≈ [Ala^9^] ≈ [Ala^6^] ≈ [Ala^2^] ≥ [Ala]^5^ ≈ [Ala^1^]uPAcyclin. These data indicate that [Ala^2^]uPAcyclin, [Ala^5^]uPAcyclin and [Ala^1^]uPAcyclin variants possess a binding affinity definitely higher than that of the parental uPAcyclin.

Alterations of the amino acid sequence may affect the peptide structure and may, in turn, alter its function. To test for differences in the inhibitory activity of the Ala-substituted peptides, U87-MG glioblastoma cells were subjected to a directional migration assay in Boyden chambers towards FBS, in the presence or in the absence of the indicated concentrations of uPAcyclin or one of its Ala-scan derivatives (Figure 1B).

As expected, uPAcyclin exhibits a dose-dependent inhibitory activity that starts at 1 pM and, at 1 µM, abolishes FBS-induced directional migration. Although [Ala^1^]uPAcyclin shows a higher affinity to the target compared to uPAcyclin, it reduces migration by 25–30%, only at a 1 µM concentration, indicating that the first residue of uPAcyclin is relevant to the peptide inhibitory activity. A comparison of the eight derivatives with the parental uPAcyclin in the inhibitory ability of cell migration confirmed that the high-affinity ligands [Ala^2^]uPAcyclin and [Ala^5^]uPAcyclin are also strong inhibitors. Regarding the other derivatives, [Ala^4^]uPAcyclin shows an inhibitory activity lower than that of uPAcyclin, in agreement with the poor binding affinity. The other derivatives, [Ala^6^]uPAcyclin, [Ala^7^]uPAcyclin, [Ala^8^]uPAcyclin and [Ala^9^]uPAcyclin, exhibit an inhibitory potential comparable to or slightly higher than that of uPAcyclin.

In contrast, substitutions of residues at positions 2, 5, 6, 7, 8 and 9 did not impair the peptide’s ability to inhibit cell migration compared to the parental uPAcyclin, showing that most residues can be Ala-replaced without a loss of activity, with the only exceptions being Lys^1^ ([Ala^1^]uPAcyclin) and Ser^4^ ([Ala^4^]uPAcyclin). Noticeably, [Ala^2^]uPAcyclin and [Ala^5^]uPAcyclin show an IC_50_ of about 10 pM, about 10-fold lower than the parental uPAcyclin (IC_50_ 100 pM). The same analogs also increased the binding affinity of about 1 log.

The [Ala^4^]uPAcyclin variant reduced binding and inhibitory abilities, suggesting that this residue is relevant for binding and that the inhibitory potential is reduced as a result of impaired binding. Concerning [Ala^1^]uPAcyclin, although the Kd_app_ is decreased compared to the uPAcyclin, this peptide lacks anti-migratory activity at 1 pM, whereas only a 30% reduction can be observed at 1 µM. An opposite behavior is exhibited by the [Ala^8^]uPAcyclin variant with a reduced affinity but a remarkable inhibitory capacity. In this case, the data suggest that this position is relevant for binding to the target integrin.

### 3.2. Conformational Analysis of Selected Peptides

Given the interesting properties exhibited by [Ala^1^]uPAcyclin, with a high affinity but low inhibitory activity, and by [Ala^5^]uPAcyclin endowed with strong inhibitory activity and a high affinity binding, they were selected for further studies. In fact, peptide [Ala^5^]uPAcyclin was selected because it showed the best affinity for an αV target in the binding assay with FITC-uPAcyclin and one of the strongest inhibitors of migration. In contrast, peptide [Ala^1^]uPAcyclin showed a high affinity but was almost unable to inhibit tumor cell migration.

To gain insights into the three-dimensional structure of these peptides, the solution conformations of [Ala^1^]uPAcyclin and [Ala^5^]uPAcyclin were analyzed via NMR. The NMR spectra of [Ala^1^]uPAcyclin in water were very similar to those of the parent uPAcyclin [18] with many signals doubled due to conformational heterogeneity. In the Supporting Information, the proton assignments of the main signal system of peptide [Ala^1^]uPAcyclin is reported (Table S1), together with the corresponding ones of uPAcyclin (Table S4) for comparison purposes. Even if we could not calculate the 3D structure of [Ala^1^]uPAcyclin due to overlapping signals from different systems, the peptide conformation must be very similar to that of uPAcyclin, and hence, the different biological behavior of [Ala^1^]uPAcyclin compared to uPAcyclin is a direct consequence of the loss of the Lys^1^ side chain in the first position. Considering that the [Ala^1^]uPAcyclin peptide variant has no reduced binding affinity but exhibits a reduced biological activity, Lys^1^ may be relevant to the inhibitory activity, whereas it is not involved in binding.

In contrast, the NMR spectral signature of [Ala^5^]uPAcyclin is very different compared to those of [Ala^1^]uPAcyclin and uPAcyclin since only one main signal pattern is observable with well-resolved signals. Complete ^1^H NMR chemical shift assignments were effectively achieved according to the Wüthrich procedure (Table S2, Supporting Information) [36]. NMR spectral features point to an extended conformation of many residues of [Ala^5^]uPAcyclin associated with strong H_α_-HN(i,i+1) signals observed in its NOESY spectrum (Table S5, Supporting Information). A medium-range NOE H_α_-HN(i,i+2) between Pro^6^ H_α_ and Glu^8^ HN, which is diagnostic of a turn, was detected together with other few medium-range NOEs, pinpointing a folded region encompassing these residues.

**Figure 2:**
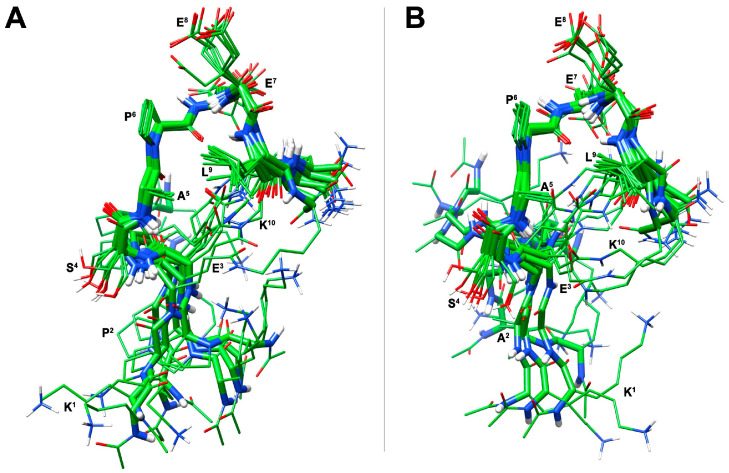
Ten lowest-energy structures of peptides [Ala^5^]uPAcyclin (**A**) and [Ala^2^, Ala^5^]uPAcyclin (**B**), as derived from NMR analyses. Structures were fitted using the heavy atoms of the cyclic residues 4–9. Heavy atoms are shown in atom-type coloring (carbon, green; nitrogen, blue; oxygen, red; hydrogen, white). For clarity, only polar hydrogen atoms are shown. The backbone-heavy atoms (residues 4–9) RMSD’s are 0.21 Å for [Ala^5^]uPAcyclin (**A**) and 0.23 Å for [Ala^2^, Ala^5^]uPAcyclin.

A structural calculation of [Ala^5^]uPAcyclin based on NMR constraints was carried out via constrained simulated annealing, and it yielded an ensemble of 10 structures (Figure 2A) satisfying the NMR-derived constraints (violations smaller than 0.10Å). The backbone of the central cyclic residues (residues 4–9) is well defined with RMSD = 0.21Å, while the N- and C-terminal regions (residues 1–3 and 10) are more flexible. Also, the side chains of the central residues are well defined. In this well-defined region, an inverse ɣ-turn is observed, centered on Glu^7^. The turn is stabilized by a hydrogen bond between the carbonyl oxygen of Pro^6^ and the amide proton of residue Glu^8^. This H-bond is in accordance with the low temperature coefficient of the last proton signal (Table S2). A cluster formed by residues Ala^5^, Pro^6^, Glu^8^ and Leu^9^ around the turn structure is also observed (Figure 2A). The finding that the substitution of Pro^5^ reduces neither the binding affinity nor inhibitory activity suggests that Pro^5^ is not directly involved in any of these interactions/activities. Rather, the Pro mutation in Ala changed the peptide conformation, thus probably disposing in a more suitable orientation the residues relevant to binding and activity.

### 3.3. Development of the Novel Peptide [Ala^2^, Ala^5^]uPAcyclin (uPAcyclin-II)

Considering the performance of Ala peptides in binding to the αV target and inhibiting cell migration, the replacement of Pro^2^ or Pro^5^ with Ala-generated cyclopeptides endowed with high affinity and high inhibitory capacities (IC_50_ around 1 pM). The two peculiar locations of exocyclic Pro^2^ and endocyclic Pro^5^ suggest that they may impact independently on the peptide general structure.

Based on these considerations, the double substitution may produce synergistic effects. To test this hypothesis, the two mutations (Pro^2^ to Ala and Pro^5^ to Ala) were combined together to provide a novel peptide [Ala^2^, Ala^5^]uPAcyclin (Ac-Lys^1^-Ala^2^-[Glu^3^-Ser^4^-Ala^5^-Pro^6^-Glu^7^-Glu^8^-Leu^9^-Lys^10^]-NH_2_).

**Figure 3:**
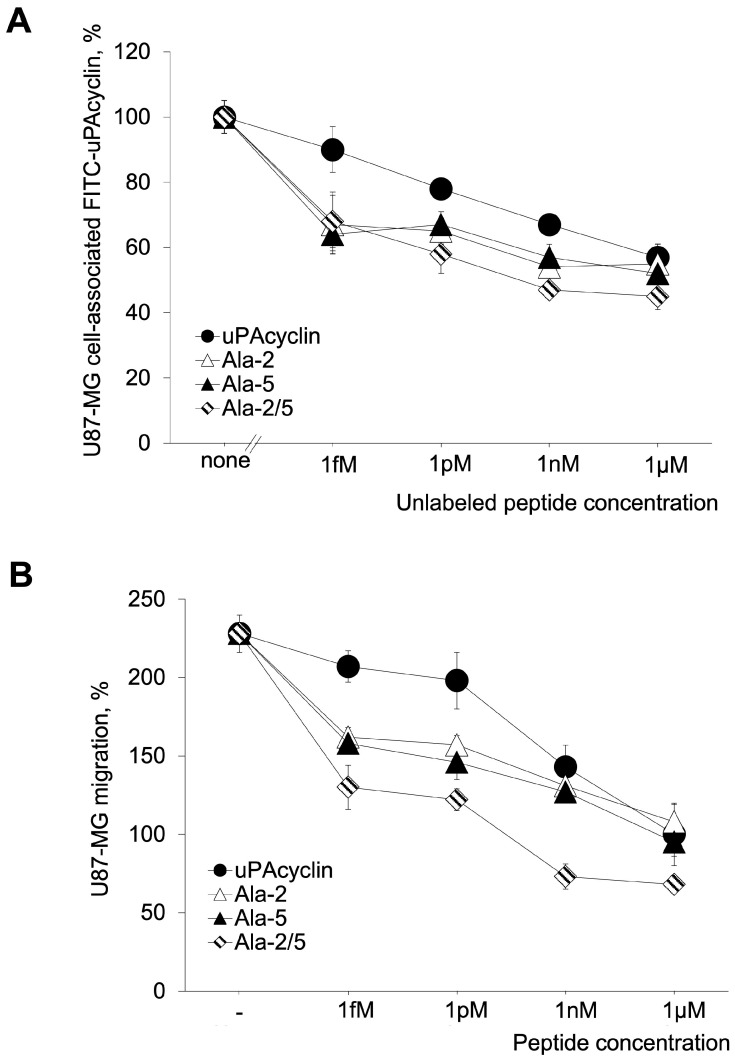
Binding and biological effect of [Ala^2^, Ala^5^]uPAcyclin on U87-MG cells. (**A**) The binding assay was performed using U87-MG cells, as described in the legend of Figure 1. The competing peptides and relative concentrations are indicated in the Figure. (**B**) The directional migration toward 5% FBS of U87-MG cells was performed in Boyden chambers, in the presence of the indicated peptides, as described in the legend of Figure 1. uPAcyclin, [Ala^2^]uPAcyclin, and [Ala^5^]uPAcyclin are also reported for comparison. For these peptides, all data points but 1 fM concentration were the same as those reported in Figure 1. For both assays, the *p*-values are in the range of 0.005 ≤ *p* ≤ 0.001.

Interestingly, [Ala^2^, Ala^5^]uPAcyclin shows an increased ability to compete with FITC-uPAcyclin in a binding assay (Figure 3A, Kd_app_ < 1 fM) and to be the most potent peptide of the series to inhibit U87-MG GBM cell migration (Figure 3B). uPAcyclin, [Ala^2^]uPAcyclin, [Ala^5^]uPAcyclin and Å6 are reported for comparison in Figure 3 and/or Figure S4 (Supporting Information). The further increased binding affinity of the Ala-2/5 variant corresponds to an approximately 1 pM IC_50_ for inhibition of cell migration, which is about 10-fold lower than the single variants and 100-fold lower than the parental uPAcyclin, which is about 1000-fold lower than Å6. It is of note that the di-substituted variant reduces migration to about 70%, thus inhibiting not only FBS-induced but also random cell migration. Thus, a comparison of the binding affinity and inhibitory potential of these cyclic peptides with the parental uPAcyclin indicated that the [Ala^2^, Ala^5^]uPAcyclin is definitely the best-performing derivative, more active than the single [Ala^2^]uPAcyclin and [Ala^5^]uPAcyclin peptides. All of these peptides are active at a lower concentration than uPAcyclin, which is our reference peptide. Interestingly, the pre-existing Å6 peptide is much less active compared to uPAcyclin and [Ala^2^, Ala^5^]uPAcyclin (Figure S4).

[Ala^2^,Ala^5^]uPAcyclin was subjected to NMR conformational analysis. The general analysis provided very similar results compared to the [Ala^5^]uPAcyclin, with the signals of the common cyclic region almost overlapping in the spectra of the two peptides (Tables S2 and S3). When considering the mutated exocyclic fragment Ac-Lys^1^-Ala^2^ of [Ala^2^,Ala^5^]uPAcyclin, all NMR parameters pointed to a random conformation, as for the corresponding region of [Ala^5^]uPAcyclin. A structure calculation for [Ala^2^, Ala^5^]uPAcyclin, based on NOE-derived constraints (Table S6), provided the conformation ensemble depicted in Figure 2B. Compared to [Ala^5^]uPAcyclin, and as expected from a qualitative inspection of the NMR data, the [Ala^2^, Ala^5^]uPAcyclin conformational preferences resemble those of peptide [Ala^5^]uPAcyclin (Figure 2A). In conclusion, the main conformational differences between the single, Ala5, and the double, Ala2/5, variants are confined within the N-terminal tails, which came out to be more flexible as a consequence of the Pro^2^ to Ala^2^ mutation. The finding that change in residues 2 and 5 reduces neither binding nor biological activity suggests that these residues are not directly involved in critical molecular interactions; in contrast, their Ala-substitutions lead to major conformation changes, as shown through the NMR analyses. Hence, the functional effects of the di-substitution in Ala2/5 are likely to be due to the described conformational changes.

### 3.4. Effects of the Relevant Peptides on the U251-MG Cell Lines

**Figure 4:**
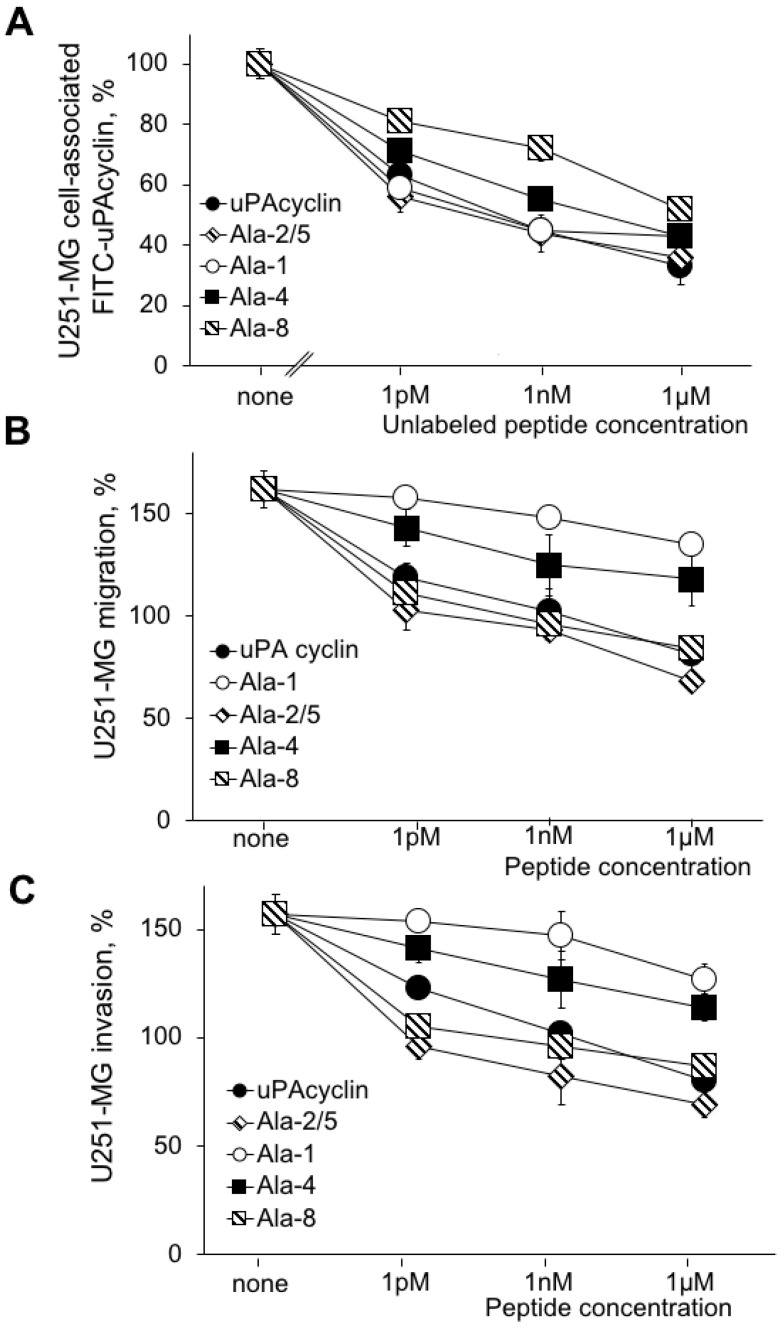
Binding and biological effect of selected Ala peptides on U251-MG migration and invasion. (**A**) A binding assay was performed using U251-MG cells, as described in the legend of Figure 1. The competing peptides and relative concentrations are indicated in the figure. (**B**) The directional migration toward 10% FBS of U251-MG cells was performed in Boyden chambers, in the presence of the indicated peptides, as described in the legend of Figure 1. (**C**) An invasion assay was performed toward 5% FBS of U251-MG cells in Boyden chambers, in the presence of the indicated peptides. For all migration and invasion assays, the *p*-values are ≤0.001, apart from [Ala^1^]uPAcyclin with a *p* ≤ 0.05.

To rule out the possibility that the effects observed may be restricted to a single cell line, testing was extended to the U251-MG cell line, derived from a human glioblastoma with a mesenchymal-like phenotype with a lower proliferative capacity than U87-MG cells. This cell line was analyzed for the ability to specifically bind the FITC-uPAcyclin, in the presence of increasing concentrations of unlabeled [Ala^2^,Ala^5^]uPAcyclin, together with the parent uPAcyclin and the selected cyclopeptides [Ala^1^]uPAcyclin, [Ala^4^]uPAcyclin and [Ala^8^]uPAcyclin (Figure 4A). In particular, [Ala^1^]uPAcyclin and [Ala^8^]uPAcyclin were selected for their contrasting affinity/activity observed on U87-MG glioblastoma cells, while [Ala^4^]uPAcyclin was chosen to confirm the importance of Ser4 both in binding and in the anti-migratory activity of uPAcyclin.

A dose-dependent decrease in cell-associated fluorescence was observed for uPAcyclin, showing the occurrence of specific binding to the U251-MG cells. As expected, the uPAcyclin inhibits migration and invasion of U251-MG cells, over the 1 pM–1 µM concentration range (Figure 4B,C).

Similarly to what was observed for U87-MG cells, the Ala^1^ variant retains the ability to target integrin, but it has little or no ability to inhibit migration and invasion. The results obtained with the U-251-MG cells are in line with those obtained with the U87-MG cells showing the peptides [Ala^2^,Ala^5^]uPAcyclin and [Ala^8^]uPAcyclin as the most active among the Ala variants of uPAcyclin. This set of experiments shows that the di-substituted Ala^2/5^ variant binds with the highest affinity and is the most potent inhibitor of U251-MG migration and invasion, with an IC_50_ less than 1 pM.

### 3.5. F-Actin Redistribution in Response to the Novel Peptides

**Figure 5:**
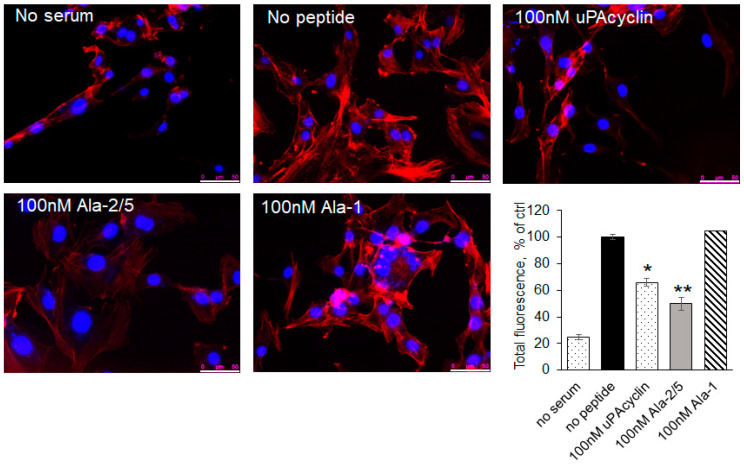
Representative images of U87-MG cytoskeletal rearrangements. Cells were pre-incubated for 40 min at 37 °C with 100 nM of uPAcyclin, [Ala^2^,Ala^5^]uPAcyclin or [Ala^1^]uPAcyclin and then allowed to adhere for 3 h in the presence of 5% FBS. Samples without serum were included as a control. F-actin was detected via staining with rhodamine–phalloidin, as specified in the Methods section, using the Leica DMI 6000 Inverted Microscope at 40× magnification. Scale bar: 50 µm. In the histogram, the results are expressed as a percentage of total fluorescence intensity in the absence of FBS and peptide (no peptide); ** *p*-value < 0.005, * *p* ≤ 0.05.

Actin reorganization plays a crucial role in cell adhesion and migration [43]. To investigate whether uPAcyclin and its relative variants affect the cell cytoskeleton, which could provide insights into the mechanism of action, the U87-MG cell distribution of F-actin was analyzed via rhodamine–phalloidin staining (Figure 5). As shown in the histogram, 5% FBS (no peptide) increased the fluorescence intensity by 5-fold with respect to the basal value (no serum). Cells’ pre-incubation with 100 nM of uPAcyclin causes a 35% reduction in total fluorescence, thus inhibiting actin reorganization. Interestingly, [Ala^2^,Ala^5^]uPAcyclin showed a greater inhibition (50%) of the cytoskeletal rearrangements, a prerequisite to cell adherence and migration. As expected, pre-exposure to [Ala^1^]uPAcyclin is ineffective. These results support the possibility that uPAcyclin derivatives, in particular the [Ala^2^,Ala^5^]uPAcyclin variant, interfere with cytoskeleton assembly and cell migration.

### 3.6. Inhibition of Vasculogenic Mimicry of U87-MG and U251-MG Cells

It is known that GBM tumors are resistant to treatments targeting the development of blood vessels. A likely possibility is that they form vessel-like structures originating from the tumor cells and not from endothelial precursors. The latter process is named vasculogenic mimicry (VM) and can be reproduced in culture [44,45]. When U87-MG or U251-MG cells are cultured under serum-free conditions in gelled extracellular matrices like GeltrexTM, they form clearcut vascular-like structures in 8–24 h. These structures develop from cell protrusions that connect to each other and form polygonal networks, resembling those formed by human endothelial cells.

**Figure 6:**
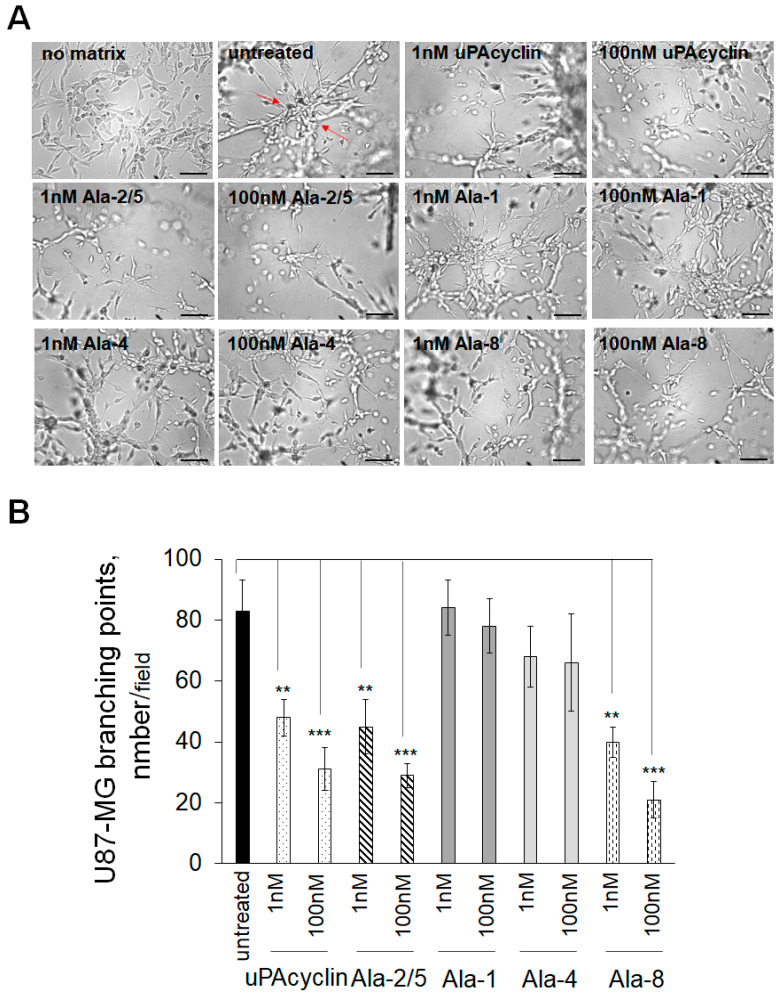
Inhibition of vasculogenic mimicry by U87-MG glioblastoma cells. (**A**) Representative images of U87-MG cells cultured in DMEM/10% FBS (no matrix) or seeded onto Geltrex^TM^ in multiwell-96 (3 × 10^4^ cells/well). When specified, cells were pre-treated with diluents (untreated) or with the indicated peptides at the indicated concentrations for 40 min at 37 °C prior to the VM assay. All images are taken after 24 h with the DMI Leica 6000 Inverted Microscope and 50× total magnification; scale bar: 100 μm. (**B**) The quantitation of branching points, defined as the intersection of at least three tubes (red arrows), was performed at 24 h either in control cells and cells exposed to the peptides and reported in the histogram (** *p*-value < 0.005; *** *p*-value < 0.001).

**Figure 7:**
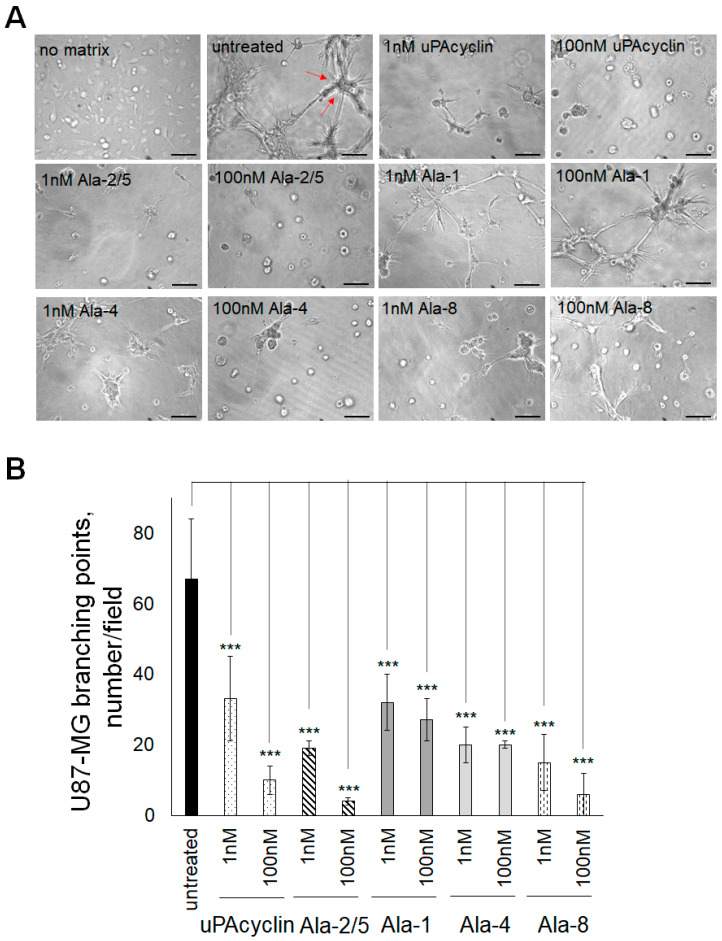
Inhibition of vasculogenic mimicry by U251-MG glioblastoma cells. (**A**) Representative images of U251-MG cells cultured in DMEM/10% FBS (no matrix) or seeded onto Geltrex^TM^ in multiwell-96 (1.5 × 10^4^ cells/well). When specified, cells were pretreated with diluents (untreated) or with the indicated peptides at the indicated concentrations for 40 min at 37 °C prior to the VM assay. All images are taken after 24 h with the DMI Leica 6000 Inverted Microscope with 50× total magnification; scale bar: 100 μm. (**B**) The quantitation and expression of branching points (red arrows), is accomplished as reported in the legend of Figure 6 (*** *p*-value < 0.001).

Here, U87-MG cells were plated onto Geltrex^TM^, and the formation of round vascular-type structures was assessed after 24 h (Figure 6A). The untreated sample shows elongated and interconnected cells exhibiting a countable number of intersections, named branching points, which are determined to quantify the extent of VM formation, highlighted in Figure 6 (untreated) by red arrows. If cells are pretreated with nanomolar concentrations of the indicated cyclic peptides for 40 min at 37 °C prior to the VM assay, the number of intersections decreases. For this assay, the peptides [Ala^2^, Ala^5^]uPAcyclin, [Ala^1^]uPAcyclin [Ala^4^]uPAcyclin and [Ala^8^]uPAcyclin, in addition to the parent uPAcyclin, were selected based on previous considerations. In the histogram shown in Figure 6B, the number of intersections per field is reported for untreated or peptide-exposed samples. As a reference, the uPAcyclin is indeed able to reduce vessel formation by 40% at 1 nM and by 70% at 100 nM. In contrast, [Ala^1^]uPAcyclin and [Ala^4^]uPAcyclin are not effective inhibitors, even at 100 nM. [Ala^2^,Ala^5^]uPAcyclin and [Ala^8^]uPAcyclin are able to reduce VM by 50% at 1 nM and by 70% at 100 nM, confirming that they are both efficient inhibitors of GBM functions supporting malignancy. The formation of VM was also monitored in U251-MG cells, which were seeded under the same conditions employed for U87-MG cells. Representative images, reported in Figure 7A, show the elongated structures obtained after 24 h in Geltrex^TM^ matrix and their reduction in the cells pre-exposed to uPAcyclin, [Ala^2^,Ala^5^]uPAcyclin and [Ala^8^]uPAcyclin. As shown in the histogram reported in Figure 7B, the uPAcyclin is indeed able to reduce vessel formation by 50% at 1 nM and by 80% at 100 nM. Furthermore, [Ala^2^,Ala^5^]uPAcyclin and [Ala^8^]uPAcyclin are slightly more effective inhibitors, whereas [Ala^1^]uPAcyclin and [Ala^4^]uPAcyclin are less potent than uPAcyclin (Figure 7A,B). It is of note that, unlike [Ala^2^,Ala^5^]uPAcyclin or [Ala^8^]uPAcyclin, [Ala^1^]uPAcyclin is ineffective, even if employed at 100 nM.

### 3.7. Effects of uPAcyclin and Ala-Peptides on Cell Proliferation

To rule out the possibility that the effects of uPAcyclin and Ala peptides could be due to the inhibition of cell proliferation, cell growth in the presence of these peptides was monitored for 24, 48 and 72 h (Supporting Information, Figure S2). U87-MG and U251-MG cells were exposed to diluents or to serum in the presence of 100 nM uPAcyclin, [Ala^1^]uPAcyclin, [Ala^4^]uPAcyclin, [Ala^8^]uPAcyclin and [Ala^2^,Ala^5^]uPAcyclin (for the peptide choice, see above), and cell viability was tested at the indicated times using Trypan blue (Figure S2A,B). Both cell lines proliferate in culture in the presence of serum (DMEM/10% FBS), but not in a serum-free medium (DMEM). The growth rates of the U87-MG and U251-MG glioblastoma cell lines were similar, regardless of the exposure to 100 nM of uPAcyclin, [Ala^1^]uPAcyclin, [Ala^4^]uPAcyclin and [Ala^8^]uPAcyclin. [Ala^2^,Ala^5^]uPAcyclin-treated U87-MG and U251-MG cells showed a slight but not significant decrease in the proliferation rate after 24 and 48 h (Figure S2A,B).

## 4. Discussion

In recent years, anti-cancer peptides have been regarded as a valid alternative strategy for cancer treatment because of their high specificity, reduced toxicity and side effects, thus holding great promise in the near future.

The urokinase-derived uPAcyclin decapeptide has anti-tumoral activities in vivo and a great anti-invasive potential for human GBM cells [18,26]. Here, further advancements in the structure–function relationship of this cyclic peptide, together with novel high-affinity Ala-substituted uPAcyclin variants and their NMR structural studies, are reported. In this study, with the exception of the residues involved in peptide cyclization, all were substituted with Ala residues. The resulting peptides were analyzed for the inhibition of GBM cell migration, invasion and the ability to form vascular structures, highlighting positions relevant to binding affinity and/or biological activity.

One of the most interesting derivatives is the [Ala^1^]uPAcyclin, carrying the mutations of Lys^1^ to Ala. In fact, Lys^1^ replacement with Ala has a detrimental effect on the anti-migratory activity, whereas it does not significantly affect binding affinity (Figure 1). Since the conformational preferences of [Ala^1^]uPAcyclin do not differ compared to the uPAcyclin, as ascertained via NMR analysis, it is likely that the dramatic decrease in activity is due to the direct involvement of this residue in eliciting the αV-dependent inhibitory response. Instead, the slight increase in binding affinity of [Ala^1^]uPAcyclin may be possibly ascribed to some electrostatic or steric bumping by Lys^1^ in the ligand–receptor interaction that is relieved by the mutation.

Opposite to the Ala-substitution of Lys^1^, Glu^8^-to-Ala^8^ mutation greatly reduces the binding affinity of [Ala^8^]uPAcyclin, and yet, it does not affect its anti-migratory activity. This suggests that Glu^8^ is directly involved in binding but is unnecessary for the biological activity of uPAcyclin. These observations lead to the conclusion that there are different requirements for binding and activity in the ligand structure, uncovering interesting tools to modulate peptide properties.

Other mutations gave rise to the coherent modulation of binding and activity. In fact, Ser^4^ to Ala mutation decreased both binding and activity, suggesting that [Ala^4^]uPAcyclin activity may fail as a result of impaired binding.

In contrast, all Pro to Ala-substitutions led to an improvement in binding and activity. In particular, Ala-substitutions of Pro^5^ led to substantial changes in the three-dimensional structure of the derivative, as assessed via NMR analyses (Figure 2A). These changes ameliorated the peptide properties, as [Ala^5^]uPAcyclin is endowed with 10-fold higher binding and inhibitory activities than parental uPAcyclin (Figure 1). As assessed through NMR studies, this variant exhibits substantial changes in the three-dimensional structure of the cyclic portion compared to uPAcyclin, possibly conferring a better orientation to residues crucial to receptor binding and/or biological activity. Similar observations also apply to the case of Ala-substituted Pro^2^ and Pro^6^, with improved performance in the binding and inhibition of GBM cells’ migration, possibly due to conformational changes.

Our studies focused on the [Ala^5^]uPAcyclin peptide, showing the best performances in the two assays. NMR analyses and NMR-based calculations of [Ala^5^]uPAcyclin provided the structures displayed in Figure 2A, where the cyclic portion of [Ala^5^]uPAcyclin is well defined. Of note, the main structural element is an inverse ɣ-turn centered on Glu^7^ and flanked by a cluster formed by the residues Ala^5^, Pro^6^, Glu^8^, and Leu^9^. The structure of [Ala^5^]uPAcyclin differs from that of uPAcyclin, characterized by a high population of the cis-isomer (>60%) at the Ser^4^-Pro^5^ amide bond [18]. This cis structure observed for uPAcyclin is probably not mandatory for biological activity since it is not found in other active linear analogs such as linear-uPAcyclin (Table 1) [18,21] and cannot be present in [Ala^5^]uPAcyclin, where Pro^5^ is replaced with Ala^5^. The conformational reorganization of [Ala^5^]uPAcyclin compared to uPAcyclin can account for its improved binding affinity and ability to inhibit tumor cell migration, invasion and vascular structure formation.

When starting from this finding and considering that the exocyclic mutation of Pro^2^ in [Ala^2^]uPAcyclin also confers an improvement to both binding and anti-migratory abilities, the mutations of Pro^2^ and Pro^5^ were combined together, aiming at obtaining a synergic effect. In line with the predictions, [Ala^2^, Ala^5^]uPAcyclin emerged as the most active peptide in the series. It significantly enhanced the affinity/activity of [Ala^2^]uPAcyclin and [Ala^5^]uPAcyclin, as well as the parent peptides uPAcyclin and Å6 (Figure 3 and Figure S1). Based on these results, [Ala^2^, Ala^5^]uPAcyclin stands out as the new lead compound for the next generation of uPAcyclins and is thus designated as uPAcyclin-II.

As expected, NMR parameters concerning the cyclic portion of [Ala^2^,Ala^5^]uPAcyclin were almost overlapping with those of [Ala^5^]uPAcyclin, and consequently, the 3D structures of the di-substituted variant strictly resemble that of [Ala^5^]uPAcyclin (Figure 2). Also, the exocyclic residues showed similar NMR parameters pointing to a random conformation for this region in both cases. Noticeably, the peculiar intrinsic higher flexibility of this region, definitely due to the Pro^2^/Ala^2^ replacement in [Ala^2^,Ala^5^]uPAcyclin, was evident in the calculated 3D structures (Figure 2B). This flexibility is likely to induce a more suitable conformation of Lys^1^ in [Ala^2^, Ala^5^]uPAcyclin to fit its biological target, justifying the observed improvement in the activity. This result agrees well with the relevant role of Lys^1^ shown through the dramatic loss of activity of [Ala^1^]uPAcyclin.

**Figure 8:**
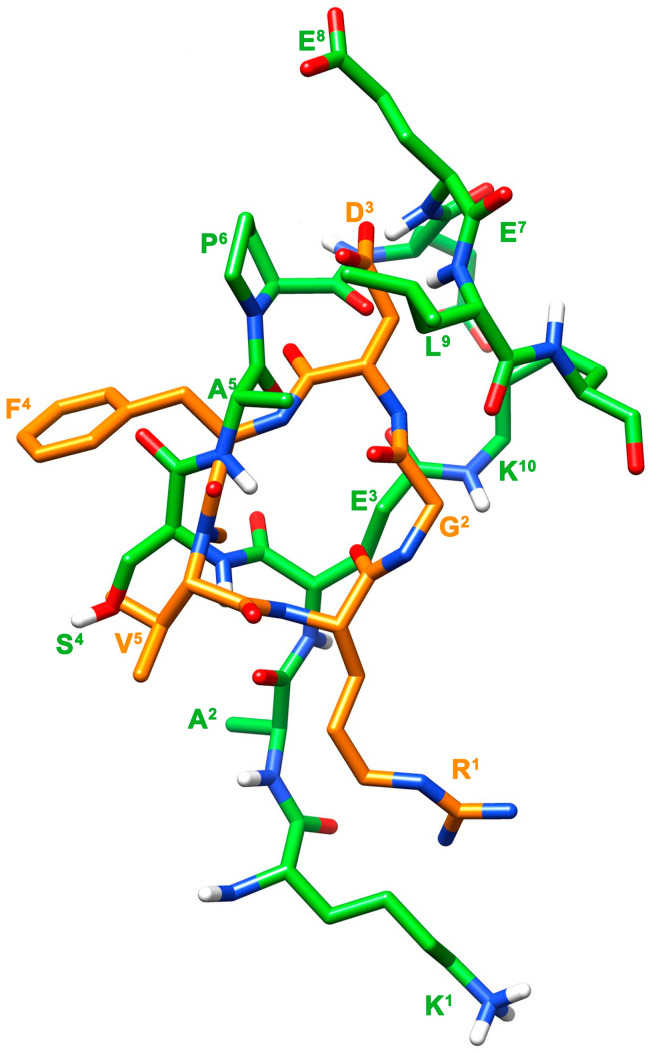
Superposition of the NMR structure of [Ala^2^, Ala^5^]uPAcyclin and the crystal structure of Cilengitide (pdb code 4MMX) [67]. The structures of [Ala^2^, Ala^5^]uPAcyclin (same color codes as in Figure 2B) and Cilengitide (orange carbon atoms) were superimposed using the heavy atoms in the following couples of side chains: Lys^1^/Arg^1^ and Glu^8^/Asp^3^.

Because extensive evidence shows that the biological target of uPAcyclin and its analogs are the αV integrins [18,26], it could be informative to compare the uPAcyclin structure with the pharmacophore of classical RGD-containing integrin ligands. Attempting to overlap the functional groups engaged (Lys vs. Arg) and pharmacophoric distances of Cilengitide, a well-known RGD peptide, generated the image shown in Figure 8. As a result, the pharmacophoric charged side chains cannot be efficiently overlapped with the putatively corresponding ones (Lys^1^ and Glu^7^ or Glu^8^) of [Ala^2^, Ala^5^]uPAcyclin, classifying uPAcyclin and its variants as non-RGD-mimetic integrin inhibitors. The data shown here are confirmatory, as [Ala^1^]uPAcyclin lacks the positively charged Lys^1^ residue, potentially mimetic of the arginine in the RGD-containing molecules; nonetheless, it is still able to bind the target integrin, even with an increased affinity.

This suggests that the binding mode of uPAcyclin to the αV target integrins does not follow the known RGD mode and needs further experimentation to be clarified. Finally, in light of the finding that the novel cyclopeptide uPAcyclin-II retains full activity, even at concentrations of 1 pM or less, it is useful to consider the active concentration range of the available integrin antagonists [46]. It is of note that the majority of integrin ligands exhibit IC_50_s in the medium-high nanomolar range, whereas few fall in the low nanomolar range, including the RGD-related Cilengitide. Whether uPAcyclin and its congeners act only on integrins or also on other receptors is to be further investigated.

Because cytoskeletal reorganization plays a crucial role in cell adhesion and migration [43], the effects of uPAcyclin, [Ala^2^, Ala^5^]uPAcyclin and [Ala^1^]uPAcyclin on the serum-induced rearrangements were investigated (Figure 5). In agreement with the binding and migration data, [Ala^2^, Ala^5^]uPAcyclin exerted a higher inhibitory effect compared to uPAcyclin, while pre-treatment with [Ala^1^]uPAcyclin is ineffective. The inhibitory effects may be secondary to the inhibition of integrin signaling, known to be connected to actin fibers in the inner cell [15]. In this case, it is possible that blocking the integrin with a specific antibody or a specific reagent could result in the inhibition of actin polymerizations. In any case, further studies are needed to investigate the mechanistic aspects of the uPAcyclin-dependent inhibition of cytoskeletal rearrangements.

Among the factors held responsible for the resistance to anti-angiogenic therapy (i.e., Bevacizumab), a central role is played by VM, an endothelial-independent vascular formation [47,48]. It represents one of the different neovascularization mechanisms, consisting of vessel-like structures originating from cancer cells [47]. VM has been described in a variety of solid tumors, including GBM, and it is associated with a poorer prognosis and higher aggressiveness [48]. Although the cellular and molecular mechanisms underlying the formation of VM are poorly understood, they are believed to arise from cancer stem cells capable of transdifferentiating into endothelial-like cells [49,50]. Notably, it is well established that GBM cancer stem cells are responsible for the maintenance of tumor growth and for its resistance to chemotherapy, radiotherapy and anti-angiogenic therapy [51]. Because the ability to undergo VM is representative of tumor aggressiveness and resistance to therapy, a number of compounds are currently being evaluated, and some of them have been demonstrated to hamper VM in preclinical models of GBM [26,42,52,53,54]. In particular, some of them, in a variety of preclinical models, exert a protecting role in healthy neurons, making them a potentially useful therapeutic option with minor harmful side effects towards normal cells surrounding the tumor mass. For example, a number of HDAC inhibitors, such as SAHA, trichostatin A (TSA), entinostat (MS275) and MC1568, have been shown to impair VM from GBM [42]. Interestingly, some of them can also play a positive role in neuroprotection, neuroplasticity and neuronal differentiation [55,56]. Similarly, the extract derived from the *Ruta graveolens* (RGWE) plant that is able to decrease the viability of human GBM cell lines to inhibit VM and neo-angiogenesis in vitro is endowed with a potent neuroprotective activity and ameliorates the ischemic damage induced in rats [54,57,58]. In this context, uPAcyclin was demonstrated to have a potent ability to inhibit vasculogenic mimicry (VM) [26]. Here, uPAcyclin-II, together with selected Ala variants, were tested. uPAcyclin-II and the analog [Ala^8^]uPAcyclin are able to inhibit the vasculogenic mimicry of glioblastoma cells at a pM concentration. Noticeably, they came out as the most potent known compounds (IC_50_ < 1 nM) to inhibit VM formation (Figure 5 and Figure 6), which makes them extremely interesting pharmacological tools and lead compounds in the development of VM contrasting agents for clinical use.

It is worth noting that other compounds proven to impair VM are also able to inhibit the expression or activation of genes or proteins involved in angiogenesis or vasculogenesis. For example, both Cilengitide and RGWE down-regulate the expression of the gene coding for VEGF-A, an essential player in angiogenesis and vasculogenesis; moreover, they both inhibit the Ras-ERK pathway, a signaling cascade essential for angiogenesis [54,59,60]. In addition, the peptide uPAcyclin is able to down-regulate VE-cadherin, a protein known to play a role in vasculogenesis, in vessel-like structures formed from GBM cells [26,61].

VM arises in a hypoxic tumor microenvironment (TME) where other cellular types, such as fibroblasts and macrophages, are known to play a role and thus might also affect VM [62]. It is worth mentioning that uPAcyclin remarkably decreased the pro-invasive ability of peritumoral CAFs from breast cancer patients and partially reversed their phenotype [18]. Interestingly, it has been shown that CAFs may foster VM formation and the expression of VM-related genes [63]. In addition, CAFs play a crucial role within the reactive stroma of the TME, engaging directly with cancer cells through secreted molecules and cell–cell adhesion and also impacting cancer cells indirectly by remodeling the extracellular matrix (ECM) and promoting immune cell infiltration [64]. Notwithstanding differences in the brain microenvironment, such as the absence of fibroblasts, it is tempting to speculate that uPAcyclin and/or its derivatives may also play a role in cells of the GBM microenvironment, such as astrocytes and/or microglia. Notably, the observation that the ability of our developed compounds to inhibit VM formation parallels their antimigratory/anti-invasive activities suggests a similar mechanism of action for these different effects. Anyway, further work will be required to address the exact mechanism(s) of action of uPAcyclin and its variants, thus identifying genes and molecules involved by means of “omic” approaches and high-throughput screening [65,66].

Finally, experiments carried out with 3D systems and/or with co-culture cellular models will clarify the contribution of different cell types and address whether the effects depend on soluble/diffusible factors or cell–cell contact [41].

## 5. Conclusions

Glioblastoma multiforme (GBM) is the most common malignant primary brain tumor, and it is characterized by high recurrency and a high mortality rate. This work has provided novel cyclopeptide analogs of uPAcyclin endowed with the ability to inhibit glioblastoma cells’ migration/invasion and microtubule formation at pM concentrations. Our work started from the determination of the residues of uPAcyclin involved in binding to αV integrins, previously demonstrated to be its biological target. A couple of residues, Ser^4^ and Glu^7^, were found to be indispensable for binding and activity, while the substitution of the prolines with Ala residues yielded variants with an improved affinity/improved activity. Interestingly enough, Lys^1^ and Glu^8^ mutations yielded conflicting results for binding and activity, indicating that there are different requirements for binding and activity in the ligand structure and uncovering interesting tools for modulating peptide properties. Through the combination of some of the obtained structure–activity relationship (SAR) information, a novel cyclopeptide, [Ala^2^,Ala^5^]uPAcyclin, uPAcyclin-II, was developed with a 100-fold improvement in binding and GBM cells’ anti-migration/invasion activity compared to uPAcyclin. An NMR conformational analysis allowed the determination of the 3D solution structure of uPAcyclin-II, which is fundamental for further cycles of optimization of this peptide class and for exploring its mechanism of action.

Extensive evidence shows that the biological target of uPAcyclin and its analogs are the αV integrins. However, both the uPAcyclin SARs and the 3D structure of its most active analogs allow the classification of uPAcyclin and its variants as non-RGD-mimetic integrin inhibitors, a newsworthy result when considering the failure of the classic RGD antagonist in clinical trials. Anyway, further work will be required to address the exact mechanism of action of uPAcyclin and its variants.

uPAcyclin-II and the analog [Ala^8^]uPAcyclin are able to inhibit the vasculogenic mimicry of glioblastoma cells at pM concentrations, which can represent a useful weapon for blocking the spread of these tumor cell. To the best of our knowledge, they are the most potent compounds inhibiting VM to date.

Taken together, our findings make the novel cyclodecapeptide uPAcyclin-II a valid lead compound in the development of novel potent anticancer agents.

## Figures and Tables

**Table 1:** Sequences of relevant peptides.

Cmpd	Sequence ^a^
linear-uPAcyclin ^b^	Ac-Lys^1^-Pro^2^-Glu^3^-Ser^4^-Pro^5^-Pro^6^-Glu^7^-Glu^8^-Leu^9^-Lys^10^-NH_2_
uPAcyclin ^b,c^	Ac-Lys^1^-Pro^2^-[Glu^3^-Ser^4^-Pro^5^-Pro^6^-Glu^7^-Glu^8^-Leu^9^-Lys^10^]-NH_2_
[Ala^1^]uPAcyclin	Ac-**Ala^1^**-Pro^2^-[Glu^3^-Ser^4^-Pro^5^-Pro^6^-Glu^7^-Glu^8^-Leu^9^-Lys^10^]-NH_2_
[Ala^2^]uPAcyclin	Ac-Lys^1^-**Ala^2^**-[Glu^3^-Ser^4^-Pro^5^-Pro^6^-Glu^7^-Glu^8^-Leu^9^-Lys^10^]-NH_2_
[Ala^4^]uPAcyclin	Ac-Lys^1^-Pro^2^-[Glu^3^-**Ala^4^**-Pro^5^-Pro^6^-Glu^7^-Glu^8^-Leu^9^-Lys^10^]-NH_2_
[Ala^5^]uPAcyclin	Ac-Lys^1^-Pro^2^-[Glu^3^-Ser^4^-**Ala^5^**-Pro^6^-Glu^7^-Glu^8^-Leu^9^-Lys^10^]-NH_2_
[Ala^6^]uPAcyclin	Ac-Lys^1^-Pro^2^-[Glu^3^-Ser^4^-Pro^5^-**Ala^6^**-Glu^7^-Glu^8^-Leu^9^-Lys^10^]-NH_2_
[Ala^7^]uPAcyclin	Ac-Lys^1^-Pro^2^-[Glu^3^-Ser^4^-Pro^5^-Pro^6^-**Ala^7^**-Glu^8^-Leu^9^-Lys^10^]-NH_2_
[Ala^8^]uPAcyclin	Ac-Lys^1^-Pro^2^-[Glu^3^-Ser^4^-Pro^5^-Pro^6^-Glu^7^-**Ala^8^**-Leu^9^-Lys^10^]-NH_2_
[Ala^9^]uPAcyclin	Ac-Lys^1^-Pro^2^-[Glu^3^-Ser^4^-Pro^5^-Pro^6^-Glu^7^-Glu^8^-**Ala^9^**-Lys^10^]-NH_2_

^a^ Ala mutations compared to uPAcyclin are highlighted in bold. ^b^ Designed from residues 136–145 of uPA [18]. ^c^ Square brackets indicate side-chain-to-side-chain cyclization.

## Data Availability

The data used to support the current study are available from the corresponding author upon reasonable request.

## Supplementary Materials

The following supporting information can be downloaded at https://www.mdpi.com/article/10.3390/cells14040259/s1: Tables S1–S4: NMR resonance assignments of selected peptides in water solution; Tables S5 and S6: NOE-derived upper limit constraints of selected peptides; Table S7: Specific interaction of FITC-conjugated uPAcyclin to U87-MG and U251-MG cell lines; Figures S1–S3: 1D ^1^H NMR and 2D NOESY spectra of selected peptides; Figure S4: Binding and biological effect of peptide Å6 on U87-MG cells; Figure S5: Cell viability assay.
