# Supplementary material for: Glioblastoma Cell Migration, Invasion and Vasculogenic Mimicry Downmodulated by Novel uPAcyclin Derivatives
Source: Cells. 2025 Feb 12;14(4):259. doi: 10.3390/cells14040259 (PMC11853379; doi:10.3390/cells14040259)
Supplement: Supplementary file 1 [file cells-14-00259-s001.zip › cells-3367924-supplementary.pdf]

SUPPORTING INFORMATION

Glioblastoma Cell Migration, Invasion and Vasculogenic Mimicry Downmodulated by Novel uPAcyclin Derivatives

Federica Santoro[1,2,$], Francesco Merlino[1,2,$], Diego Brancaccio[1], Iolanda Camerino[3], Stefania Belli[4], Amelia Cimmino[4], Paolo Grieco[1,2], Luca Colucci-D'Amato[3,5], M. Patrizia Stoppelli[4,6] Paola Franco[4,^] and Alfonso Carotenuto[1,2,^,*]

[1] Department of Pharmacy, University of Naples Federico II, 80131 Naples, Italy;
[2] Centro Interuniversitario di Ricerca sui Peptidi Bioattivi "Carlo Pedone" (CIRPeB), University of Naples Federico II, 80134 Naples, Italy;
[3] Department of Environmental, Biological and Pharmaceutical Sciences and Technologies, University of Campania "Luigi Vanvitelli", 81100 Caserta, Italy;
[4] Institute of Genetics and Biophysics "A. Buzzati Traverso" (IGB-ABT), National Research Council 80131 Naples, Italy;
[5] InterUniversity Center for Research in Neurosciences (CIRN), Naples, Italy;
[6] UniCamillus-Saint Camillus International University of Health Sciences, 00131 Rome, Italy;

* Correspondence: alfonso.carotenuto@unina.it (A.C.); Tel.: +39-081678626 (A.C.);
$ These authors contributed equally to this work as co-first.
^ These authors contributed equally to this work as co-last.

Table S1. NMR resonance assignments of main conformer of [Ala^1]uPAcyclin in water solution. p. 2

Table S2. NMR resonance assignments of main conformer of [Ala^5]uPAcyclin in water solution. p. 3

Table S3. NMR resonance assignments of main conformer of [Ala^2,Ala^5]uPAcyclin in water solution. p. 4

Table S4. NMR resonance assignments of main conformer of uPAcyclin in water solution. p. 5

Table S5. NOE derived upper limit constraints of peptide [Ala^5]uPAcyclin. p. 6

Table S6. NOE derived upper limit constraints of peptide [Ala^2,Ala^5]uPAcyclin. p. 8

Table S7. Specific interaction of FITC-conjugated uPAcyclin to U87-MG and U251-MG cell lines. p. 10

Figure S1. 1D ^{1}H NMR and 2D NOESY spectra of [Ala^1]uPAcyclin. p. 11

Figure S2. 1D ^{1}H NMR and 2D NOESY spectra of [Ala^5]uPAcyclin. p. 12

Figure S3. 1D ^{1}H NMR and 2D NOESY spectra of [Ala^2,Ala^5]uPAcyclin. p. 13

Figure S4. Binding and biological effect of peptide Å6 on U87-MG cells. p. 14

Figure S5. Cell Viability Assay. p. 15

Table S1. NMR resonance assignments[a] of main conformer of **[Ala[1]]uPAcyclin** in water solution.

Residue	NH ($^{3}J_{\alpha N}$, $-\Delta\delta/\Delta T$)[b]	$C^{\alpha}H$	$C^{\beta}H$	Others
Ala[1]	8.38 (6.6, 8.7)	4.53	1.31	
Pro[2]		4.39	1.85, 2.28	2.01(γ); 3.61, 3.81(δ)
cGlu[3]	8.49 (6.3, 7.3)	4.24	1.99	2.31, 2.39 (γ)
Ser[4]	8.38 (7.4, 9.5)	4.49	3.56, 3.67	
Pro[5]		5.03	1.74, 2.48	1.94, 2.07 (γ); 3.57, 3.53 (δ)
Pro[6]		4.33	1.84, 2.38	2.10, 1.84 (γ); 3.59, 3.87(δ)
Glu[7]	8.79 (5.9, 5.7)	4.28	1.99	2.33, 2.38 (γ)
Glu[8]	8.57 (6.1, 6.2)	4.22	1.93, 2.00, 2.00	2.37 (γ)
Leu[9]	8.31 (7.2, 8.3)	4.32	1.65	1.57 (γ); 0.92, 0.87 (δ)
cLys[10]	8.14 (7.2, 8.4)	4.20	1.75	1.33 (γ); 1.41 (δ); 3.09, 3.30 (ε); 8.09 (ζ)

[a] Obtained at pH = 5.5, and 5° C with TSP (δ 0.00 ppm) as reference shift. Chemical shifts are accurate to ±0.02 ppm.
[b] $^{3}J_{\alpha N}$ coupling constants in Hz-$\Delta\delta/\Delta T$ = temperature coefficients (ppb/K) calculated in the range 5-25 °C. Further signals: $C\underline{H_3}CO$, 1.97 ppm; $CON\underline{H_2}$, 7.18, 7.50 ppm.

Table S2. NMR resonance assignments[a] of **[Ala^5]uPAcyclin** in water solution.

Residue	NH ($^3J_{\alpha N}$, -Δδ/ΔT)[b]	$C^{\alpha}H$	$C^{\beta}H$	Others
Lys[1]	8.40 (6.7, 8.8)	4.55	1.68, 1.78	1.46(γ); 1.71 (δ); 2.99(ε); 7.60 (ζ)
Pro[2]		4.41	2.29, 1.87	2.02(γ); 3.86, 3.62(δ)
cGlu[3]	8.72 (6.9, 9.3)	4.34	2.03, 2.07	2.31, 2.36 (γ)
Ser[4]	8.61 (7.4, 6.4)	4.37	3.81, 3.91	
Ala[5]	8.11 (6.8, 8.0)	4.67	1.33	
Pro[6]		4.37	2.32, 1.96	2.04 (γ); 3.77, 3.66 (δ)
Glu[7]	8.65 (5.4, 7.5)	4.14	2.07, 2.15	2.49 (γ)
Glu[8]	8.39 (6.8, 3.2)	4.23	2.07	2.46 (γ)
Leu[9]	8.11 (7.0, 6.2)	4.38	1.64	1.59 (γ); 0.93, 0.88 (δ)
cLys[10]	8.05 (7.4, 7.0)	4.17	1.75, 1.80	1.35, 1.40 (γ); 1.44, 1.53 (δ); 3.18 (ε); 8.10 (ζ)

[a] Obtained at pH = 5.5, , and 5° C with TSP (δ 0.00 ppm) as reference shift. Chemical shifts are accurate to ±0.02 ppm.
[b] $^3J_{\alpha N}$ coupling constants in Hz-Δδ/ΔT = temperature coefficients (ppb/K) calculated in the range 5-25 °C. Further signals: C<u>H$_3$</u>CO, 1.98 ppm; CON<u>H$_2$</u>, 7.20, 7.56 ppm.

Table S3. NMR resonance assignments[a] of **[Ala^2,Ala^5]uPAcyclin** in water solution.

Residue	NH ($^3J_{\alpha N}$, -$\Delta\delta/\Delta T$)[b]	C$^\alpha$H	C$^\beta$H	Others
Lys[1]	8.23 (6.6, 7.8)	4.26	1.72, 1.79	1.45(γ); 1.72(δ); 3.01(ε); 7.60 (ζ)
Ala[2]	8.38 (6.7, 8.6)	4.30	1.37	
cGlu[3]	8.39 (7.3, 9.3)	4.37	2.08, 2.03	2.32, 2.38(γ)
Ser[4]	8.49 (7.4, 6.1)	4.40	3.84, 3.92	
Ala[5]	7.95 (6.6, 7.8)	4.69	1.34	
Pro[6]		4.39	2.33, 1.96	2.04(γ); 3.67, 3.79(δ)
Glu[7]	8.48 (5.8, 7.8)	4.15	2.08, 2.17	2.50(γ)
Glu[8]	8.29 (6.1, 3.5)	4.25	2.09	2.47(γ)
Leu[9]	7.99 (7.2, 6.2)	4.39	1.65	1.60(γ); 0.94, 0.88 (δ)
cLys[10]	7.91 (7.3, 7.3)	4.19	1.77, 1.81	1.35, 1.41 (γ); 1.44, 1.53 (δ); 3.14, 3.19 (ε); 8.09(ζ)

[a] Obtained at pH = 5.5, , and 5° C with TSP (δ 0.00 ppm) as reference shift. Chemical shifts are accurate to ±0.02 ppm.
[b] $^3J_{\alpha N}$ coupling constants in Hz-$\Delta\delta/\Delta T$ = temperature coefficients (ppb/K) calculated in the range 5-25 °C. Further signals: C$\underline{H_3}$CO, 1.99 ppm; CON$\underline{H_2}$, 7.08, 7.44 ppm.

Table S4. NMR resonance assignments of main conformer of **uPAcyclin**.*

Residue	NH ($^3J_{\alpha N}$, $-\Delta\delta/\Delta T$)^b	C$^\alpha$H	C$^\beta$H	Others
Lys^1	8.40 (6.9, 9.7)	4.59	1.71, 1.81	1.49(γ); 1.71(δ); 2.99(ε); 7.60 (ζ)
Pro^2		4.43	2.32, 1.88	2.03(γ); 3.88, 3.63(δ)
cGlu^3	8.53 (7.3, 9.4)	4.29	2.07, 1.99	2.36, 2.44(γ)
Ser^4	8.36 (8.9, 9.1)	4.52	3.60, 3.70	
Pro^5		5.06	2.08, 2.54	1.78, 1.97(γ); 3.56 (δ)
Pro^6		4.37	2.44, 1.87	2.13(g); 3.91, 3.62(d)
Glu^7	8.90 (5.8, 4.8)	4.24	2.06, 1.98	2.29(g)
Glu^8	8.67 (5.8, 7.2)	4.18	2.05, 1.94	2.25, 2.33(g)
Leu^9	8.23 (7.1, 6.8)	4.34	1.64	1.59(g); 0.96, 0.89 (d)
cLys^{10}	8.15 (7.4, 9.2)	4.21	1.78, 1.82	1.40,1.49 (γ); 1.55 (d); 3.08,3.38 (ε); 8.16 (ζ)

* Reported in Reference [9]

Table S5. NOE derived upper limit constraints of peptide **[Ala⁵]uPAcyclin**.

1	LYS	HN	1	LYS	HB2	3.92
1	LYS	HN	1	LYS	HB3	3.92
1	LYS	HN	1	LYS	QB	3.18
1	LYS	HN	1	LYS	QG	6.38
1	LYS	HA	2	PRO	HD2	3.67
1	LYS	HA	2	PRO	HD3	3.67
1	LYS	HA	2	PRO	QD	3.04
2	PRO	HA	3	cGLU	HN	2.46
3	cGLU	HN	3	cGLU	HB2	3.11
3	cGLU	HN	3	cGLU	HB3	3.11
3	cGLU	HN	3	cGLU	HG2	4.51
3	cGLU	HN	3	cGLU	HG3	4.51
3	cGLU	HN	3	cGLU	QG	3.90
3	cGLU	HA	3	cGLU	QG	3.76
3	cGLU	HA	4	SER	HN	2.68
3	cGLU	HG2	10	cLYS	HZ	3.61
3	cGLU	HG3	10	cLYS	HZ	3.61
4	SER	HN	4	SER	HB2	3.61
4	SER	HN	4	SER	HB3	3.61
4	SER	HN	5	ALA	HN	2.80
4	SER	HA	5	ALA	HN	3.33
4	SER	HB2	5	ALA	HN	4.04
4	SER	HB3	5	ALA	HN	4.04
4	SER	QB	5	ALA	HN	3.50
5	ALA	HN	5	ALA	QB	3.99
5	ALA	HN	6	PRO	HD3	5.47
5	ALA	HA	6	PRO	HD2	3.11
5	ALA	HA	6	PRO	HD3	3.30
5	ALA	QB	6	PRO	HD2	5.26
5	ALA	QB	6	PRO	HD3	4.67
5	ALA	QB	8	GLU	QB	7.40
5	ALA	QB	8	GLU	QG	7.40
5	ALA	QB	9	LEU	QB	7.31
6	PRO	HA	7	GLU	HN	2.59
6	PRO	HA	8	GLU	HN	4.04
6	PRO	HB2	7	GLU	HN	3.86
6	PRO	HB2	8	GLU	QG	6.38
6	PRO	HB3	7	GLU	HN	3.86
6	PRO	HD3	7	GLU	HN	5.50
6	PRO	HD3	9	LEU	QB	6.38
6	PRO	HD3	9	LEU	QQD	6.43
7	GLU	HN	7	GLU	HB2	3.11
7	GLU	HN	7	GLU	HB3	3.39
7	GLU	HN	7	GLU	QG	5.63
7	GLU	HN	8	GLU	HN	3.27
7	GLU	HA	8	GLU	HN	3.17
8	GLU	HN	8	GLU	QB	4.14
8	GLU	HN	8	GLU	QG	5.69
8	GLU	HN	9	LEU	HN	3.45
8	GLU	HA	9	LEU	HN	3.02
8	GLU	QB	9	LEU	HN	4.45
8	GLU	QG	9	LEU	HN	6.38
9	LEU	HN	9	LEU	HG	4.20
9	LEU	HN	9	LEU	QD1	6.53
9	LEU	HN	9	LEU	QD2	6.53

9	LEU	HA	10	cLYS	HN	2.90
9	LEU	QB	10	cLYS	HN	5.11
9	LEU	HG	10	cLYS	HN	5.50
10	cLYS	HN	10	cLYS	HB2	3.61
10	cLYS	HN	10	cLYS	HB3	3.61
10	cLYS	HN	10	cLYS	QB	3.06
10	cLYS	HN	10	cLYS	HG2	4.54
10	cLYS	HN	10	cLYS	HG3	4.54
10	cLYS	HN	11	CNH2	HN1	4.76
10	cLYS	HA	10	cLYS	HD2	5.19
10	cLYS	HA	10	cLYS	HD3	5.19
10	cLYS	HA	10	cLYS	QD	4.49

Table S6. NOE derived upper limit constraints of peptide **[Ala^{2},Ala^{5}]uPAcyclin**.

1	LYS	HN	1	LYS	HB2	4.07
1	LYS	HN	1	LYS	HB3	4.07
1	LYS	HN	1	LYS	QB	3.43
1	LYS	HN	1	LYS	QG	6.38
1	LYS	HA	2	ALA	HN	2.62
1	LYS	HB2	2	ALA	HN	4.38
1	LYS	HB3	2	ALA	HN	4.38
1	LYS	QB	2	ALA	HN	3.78
2	ALA	HA	3	cGLU	HN	2.55
3	cGLU	HN	3	cGLU	HB2	3.33
3	cGLU	HN	3	cGLU	HB3	3.33
3	cGLU	HN	3	cGLU	HG2	4.63
3	cGLU	HN	3	cGLU	HG3	4.63
3	cGLU	HN	3	cGLU	QG	4.05
3	cGLU	HA	3	cGLU	HG2	4.20
3	cGLU	HA	3	cGLU	HG3	4.20
3	cGLU	HA	3	cGLU	QG	3.51
3	cGLU	HA	4	SER	HN	2.55
3	cGLU	HG2	10	cLYS	HZ1	3.52
3	cGLU	HG3	10	cLYS	HZ1	3.52
4	SER	HN	4	SER	HB2	3.55
4	SER	HN	4	SER	HB3	3.55
4	SER	HN	4	SER	QB	3.07
4	SER	HN	5	ALA	HN	2.83
4	SER	HA	5	ALA	HN	3.30
4	SER	HB2	5	ALA	HN	4.07
4	SER	HB3	5	ALA	HN	4.07
4	SER	QB	5	ALA	HN	3.54
5	ALA	HN	5	ALA	QB	4.02
5	ALA	HN	6	PRO	HD2	5.50
5	ALA	HN	6	PRO	HD3	5.50
5	ALA	HA	6	PRO	HD2	3.33
5	ALA	HA	6	PRO	HD3	3.33
5	ALA	HA	6	PRO	QD	2.86
5	ALA	QB	6	PRO	HD2	5.29
5	ALA	QB	6	PRO	HD3	5.29
5	ALA	QB	6	PRO	QD	4.45
5	ALA	QB	8	GLU	QB	7.28
5	ALA	QB	8	GLU	QG	7.28
5	ALA	QB	9	LEU	QB	7.24
6	PRO	HA	7	GLU	HN	2.65
6	PRO	HA	8	GLU	HN	4.01
6	PRO	HB2	7	GLU	HN	3.98
6	PRO	HB3	7	GLU	HN	3.98
6	PRO	QB	7	GLU	HN	3.49
6	PRO	QD	7	GLU	HN	5.03
6	PRO	QD	9	LEU	QB	6.16
6	PRO	QD	9	LEU	QQD	6.08
7	GLU	HN	7	GLU	HB2	3.45
7	GLU	HN	7	GLU	HB3	3.45
7	GLU	HN	7	GLU	QB	2.95
7	GLU	HN	7	GLU	QG	5.66
7	GLU	HN	8	GLU	HN	3.21
7	GLU	HA	8	GLU	HN	3.17
8	GLU	HN	8	GLU	QB	4.05
8	GLU	HN	8	GLU	QG	5.73

8	GLU	HN	9	LEU	HN	3.33
8	GLU	HA	9	LEU	HN	2.99
8	GLU	QB	9	LEU	HN	4.58
8	GLU	QG	9	LEU	HN	6.38
9	LEU	HN	9	LEU	HG	4.20
9	LEU	HN	9	LEU	QD1	6.53
9	LEU	HN	9	LEU	QD2	6.53
9	LEU	HA	10	cLYS	HN	2.86
9	LEU	QB	10	cLYS	HN	5.11
9	LEU	HG	10	cLYS	HN	5.50
10	cLYS	HN	10	cLYS	HB2	3.58
10	cLYS	HN	10	cLYS	HB3	3.58
10	cLYS	HN	10	cLYS	QB	3.05
10	cLYS	HN	10	cLYS	HG2	4.57
10	cLYS	HN	10	cLYS	HG3	4.57
10	cLYS	HN	11	CNH2	HN1	4.69

Table S7. Specific interaction of FITC-conjugated uPAcyclin to both U87-MG and U251-MG cell lines

**Cell Line**	**FITC-uPAcyclin net binding, cpm**
U87-MG	3.18 10^3 +/- 0.30 10^3
U251-MG	5.24 10^3 +/- 0.53 10^3
HEK-293/αV	5.65 10^3 +/- 0.48 10^3
HEK-293	0.87 10^3 +/- 0.24 10^3

A total of 2 x 10^6 U87-MG, U251-MG, U138-MG, HEK-293/αV, or HEK-293 cells were pre-incubated for 30 min at 4 °C with 500 nM uPAcyclin and further exposed to 50 nM FITC-uPAcyclin for 2 h at 4 °C. At the end of the incubation, the unbound material was washed and cell-associated FITC-uPAcyclin was quantitated by a fluorimeter. The net value of cell-surface-associated fluorescence, corresponding to the specific binding, is shown.

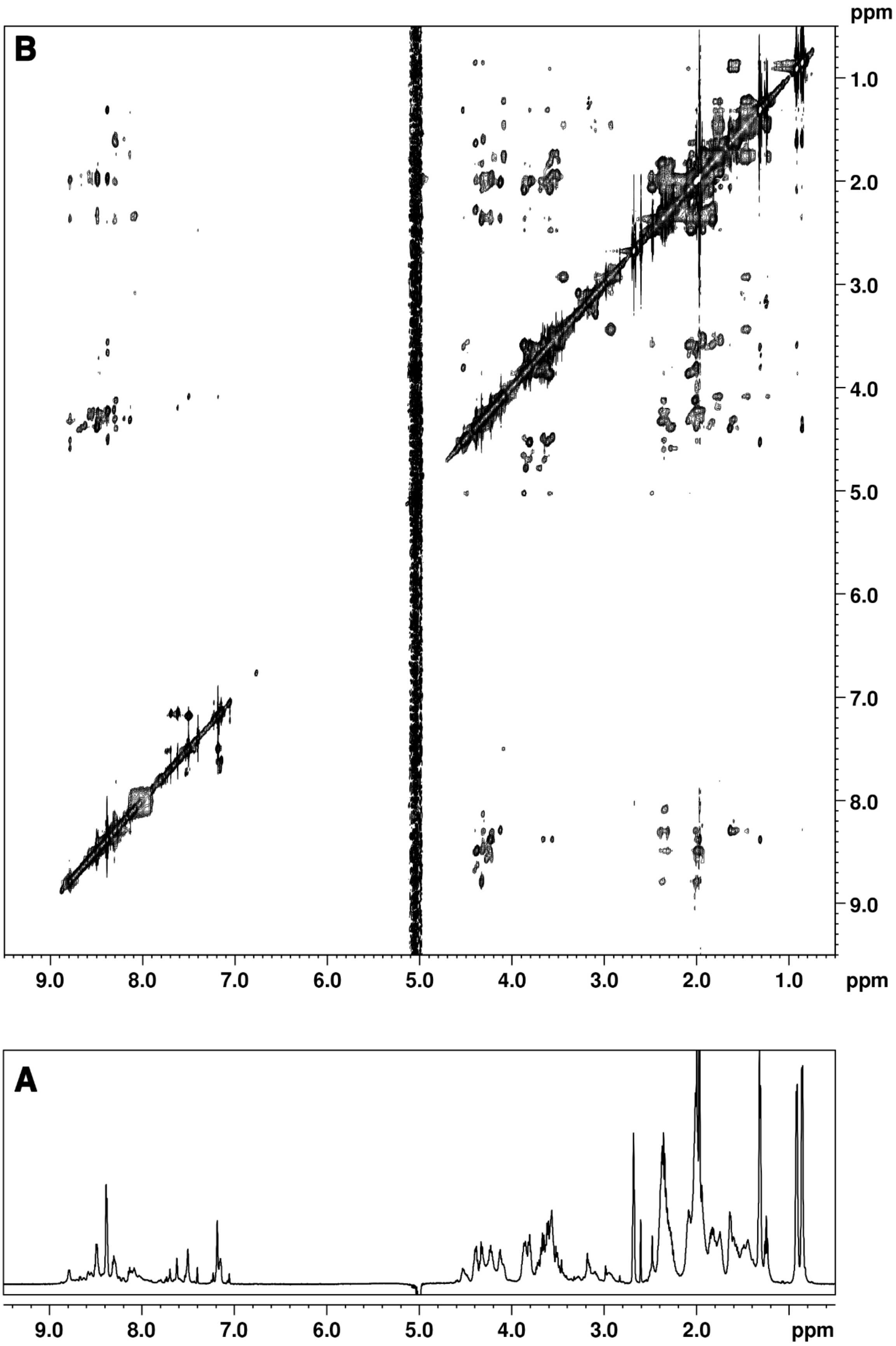

Figure S1. 1D ^{1}H NMR (A) and 2D NOESY (B) spectra of [Ala[1]]uPAcyclin.

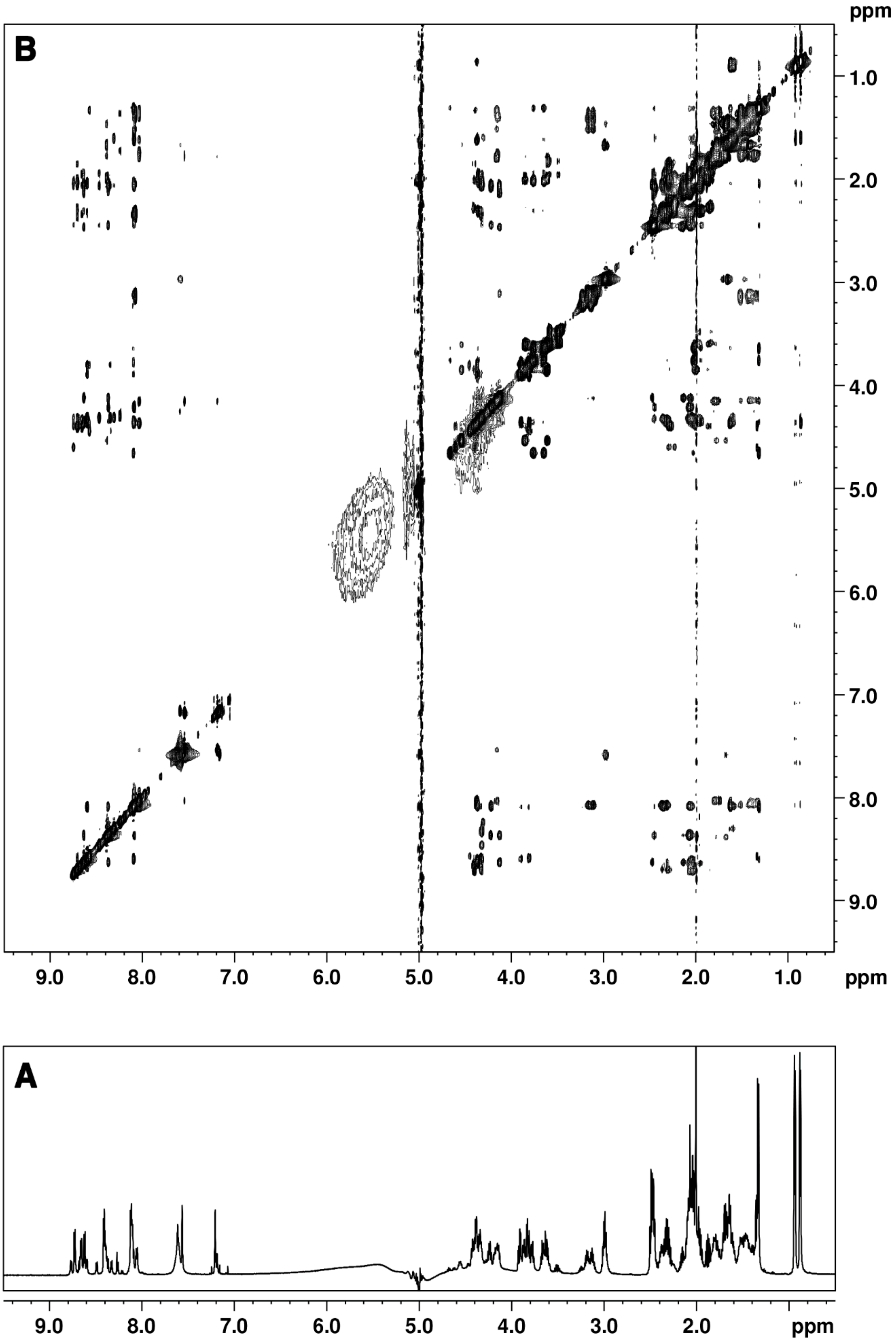

Figure S2. 1D ^{1}H NMR (A) and 2D NOESY (B) spectra of [Ala^5]uPAcyclin.

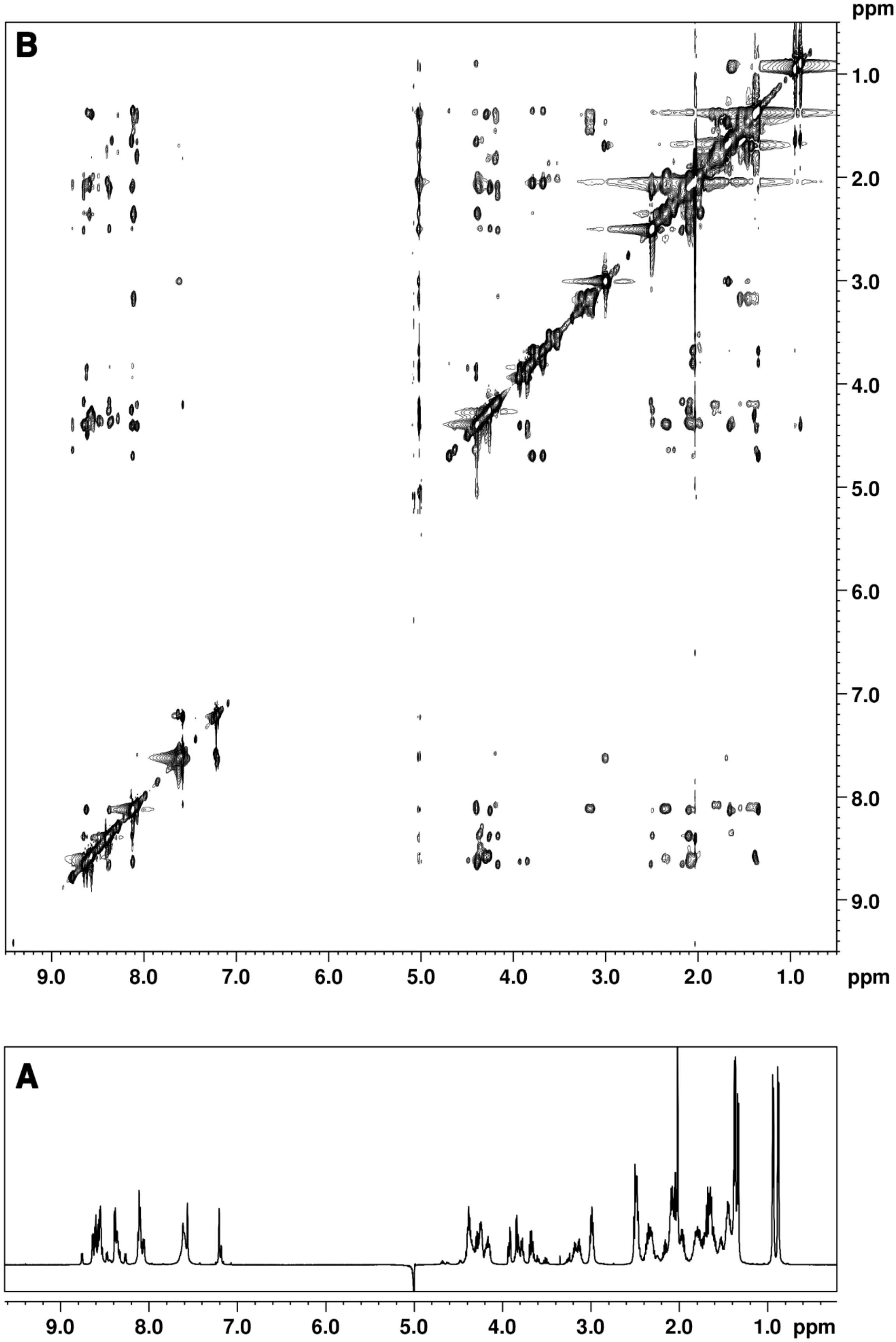

Figure S3. 1D ^{1}H NMR (A) and 2D NOESY (B) spectra of [Ala^2, Ala^5]uPAcyclin.

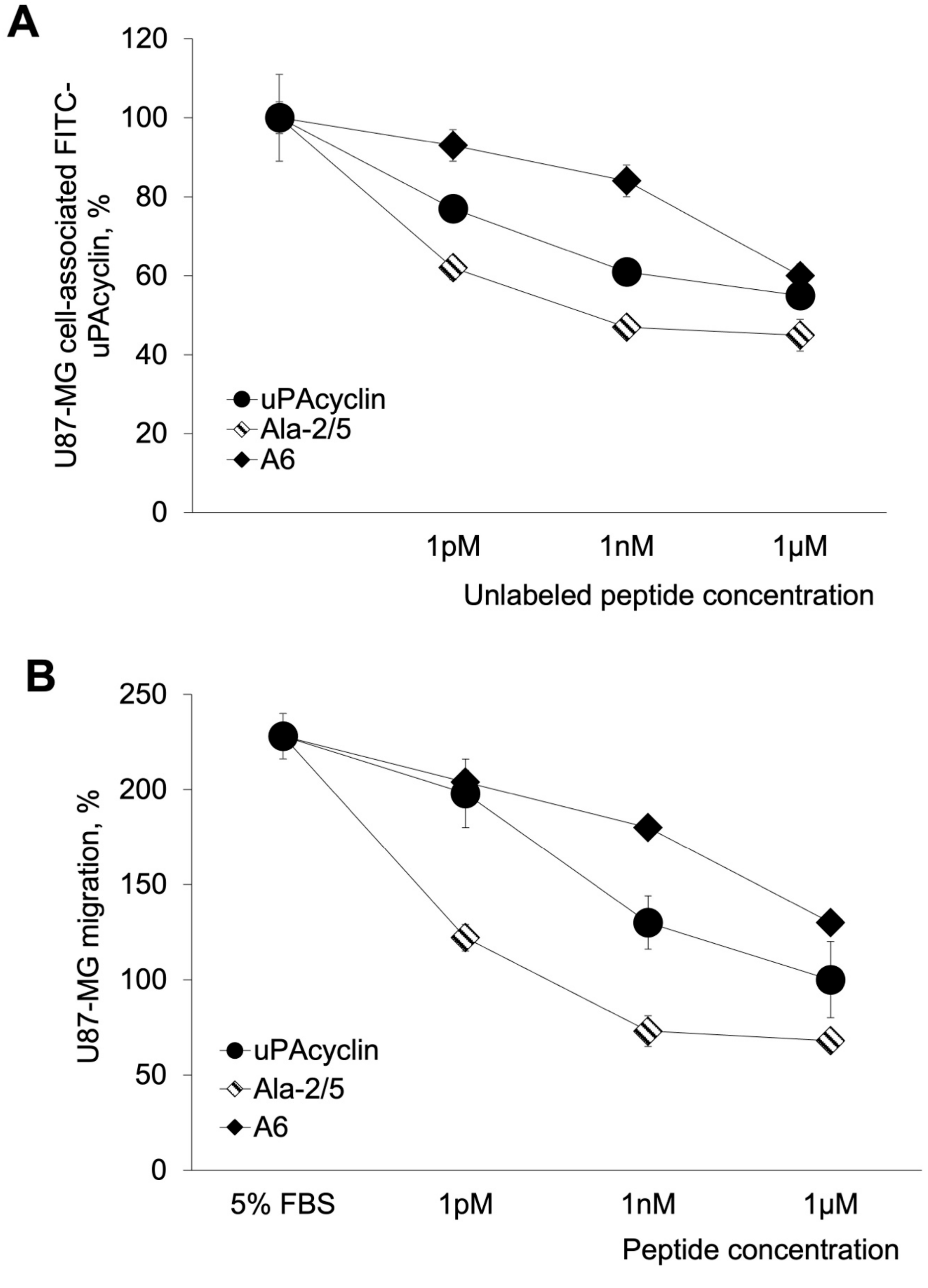

Figure S4. Binding and biological effect of peptide Å6 on U87-MG cells.

(A) Binding assay was performed using U87-MG cells as described in the legend to Figure 1. The competing peptides and relative concentrations are indicated in the Figure. (**B)** Directional migration toward 5% FBS of U87-MG cells was performed in Boyden chambers, in the presence of the indicated peptides, as described in the legend to Figure 1. uPAcyclin and [Ala^2, Ala^5]uPAcyclin data were already reported in Figure 3 and are shown here for comparison.

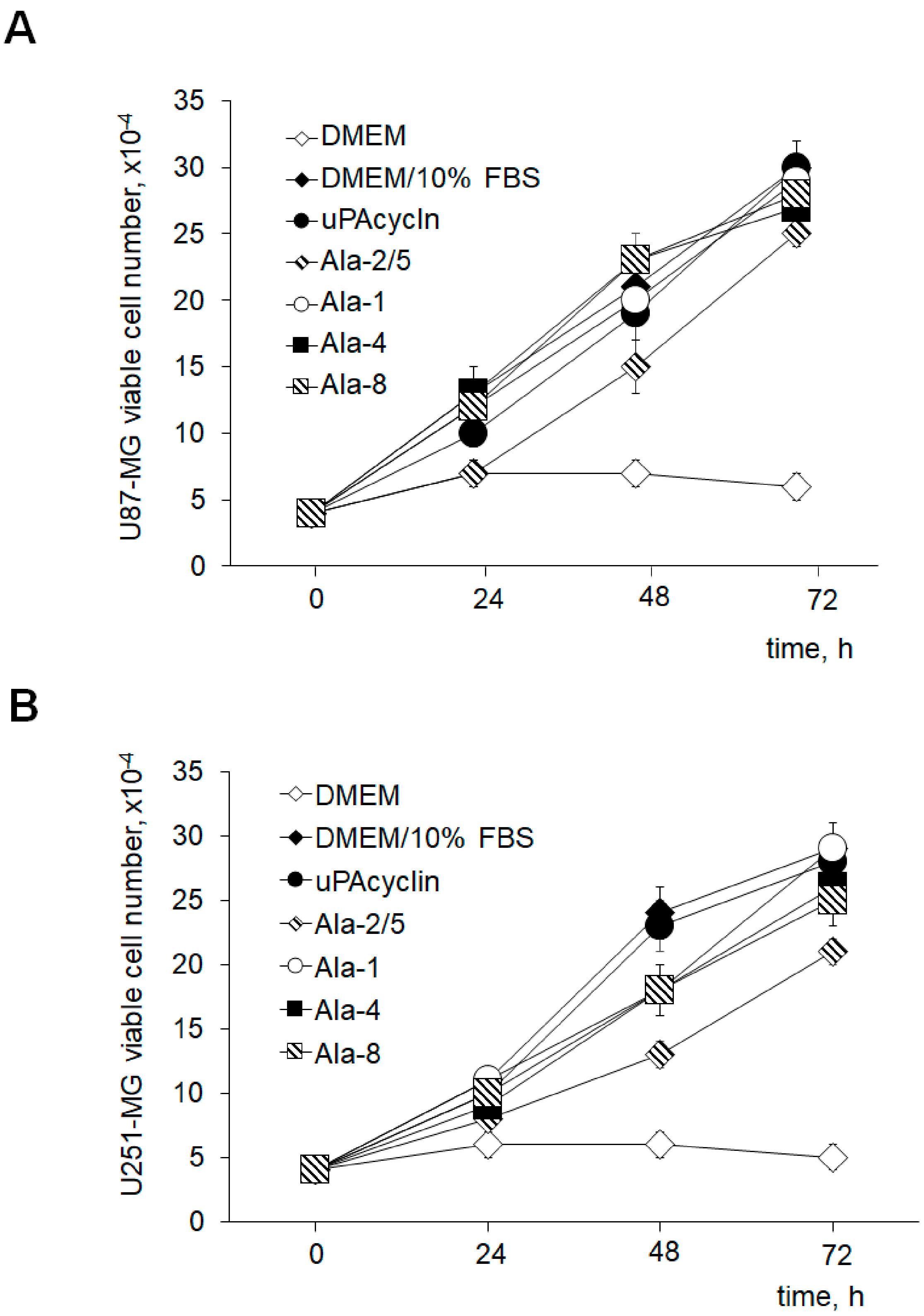

Figure S5. Cell Viability Assay.

Unaffected proliferation of U87-MG and U251-MG glioblastoma cells exposed to uPacyclin and selected Ala-peptides. 2x10^4 U87-MG (**A**) or U251-MG (**B**) were seeded in 12 well plates for 24 h in DMEM-10% FBS and serum-starved for 24 h. Then, cells were counted (0 h) and grown in DMEM (no serum) or DMEM-10% FBS, in the presence of 100 nM peptides for 24, 48 and 72 h. Histograms represent the average of two separate experiments undertaken in duplicate.
